# Novel therapeutic strategies targeting mitochondrial quality control for metabolic dysfunction-associated steatotic liver disease

**DOI:** 10.3389/fphys.2026.1803228

**Published:** 2026-06-11

**Authors:** Huiling Zuo, Jiaxin Chen, Yuhang Jiao, Xuan Ma, Xiangqiong Li, Yahui Xiao, Jiamei He, Zhuangzhuang Jia, Anhua Shi

**Affiliations:** 1 School of Basic Medical Sciences, Yunnan University of Chinese Medicine, Kunming, China; 2 Yunnan Key Laboratory of Integrated Traditional Chinese and Western Medicine for Chronic Disease in Prevention and Treatment, Kunming, China; 3 Key Laboratory of Microcosmic Syndrome Differentiation, Yunnan University of Chinese Medicine, Kunming, China

**Keywords:** metabolic dysfunction-associated steatotic liver disease (MASLD), mitochondrial, mitochondrial dysfunction, mitochondrial quality control (MQC), therapeutic strategies

## Abstract

Epidemiological evidence demonstrates that metabolic dysfunction-associated steatotic liver disease (MASLD) has evolved into one of the most widespread chronic liver disorders globally, posing a serious public health challenge. From a mechanistic perspective, the initiation of MASLD is predominantly fueled by multiple factors. Multiple pathological processes, including insulin resistance, oxidative stress, and inflammatory response, are all closely associated with the core issue of mitochondrial dysfunction. Mitochondria, serving as the hub for cellular energy metabolism, exhibit dysfunction that is widely recognized as the key nexus underlying the initiation and progression of MASLD. Maintaining mitochondrial homeostasis is the core mission of the mitochondrial quality control (MQC) system. The MQC system maintains mitochondrial homeostasis by the precise modulation of pathways including mitochondrial biogenesis (MB), mitochondrial dynamics (fusion and fission), and mitophagy. Hence, dysregulation of the MQC system may promote the pathological progression of MASLD. During the MASLD process, continuous metabolic stress disrupts the balance of MQC, and the dysregulation of MQC further exacerbates hepatocyte lipotoxicity damage, forming a vicious cycle. This review elucidates the mechanisms of MQC in MASLD as well as the latest findings. At the same time, we analyze in depth the regulatory mechanisms of each component of MQC and further explored therapeutic strategies of targeting mitochondria.

## 1 Introduction

Metabolic dysfunction-associated steatotic liver disease (MASLD), previously termed nonalcoholic fatty liver disease (NAFLD), ranks among the most prevalent chronic hepatic disorders across the globe (Rinella et al., 2023). The progression of MASLD is characterized by complexity and a protracted course, progressing from simple steatosis to metabolic dysfunction-associated steatohepatitis (MASH), and may further progress to liver cirrhosis and hepatocellular carcinoma. The onset of MASLD bears a close correlation with unhealthy lifestyle habits such as high-calorie diets and sedentary behaviors. As global lifestyle patterns shift, the incidence of MASLD sustains an upward trend. However, the complex pathogenesis has not been fully elucidated, and the classic “two-hit” hypothesis can no longer fully explain its intricate pathological process. In recent years, the “multiple strikes” hypothesis of MASLD triggered by the synergistic effects of multiple factors such as mitochondrial dysfunction, endoplasmic reticulum (ER) stress, insulin resistance, and oxidative stress has gradually gained recognition. This hypothesis suggests that the interaction and cascade reaction of multiple pathological mechanisms ultimately lead to liver cell damage and disease progression of MASLD (Friedman et al., 2018).

Within the complex pathological network of MASLD, mitochondrial dysfunction has been recognized as a pivotal mechanism underlying disease progression (Sheldon et al., 2019). Mitochondria, as the metabolic hub of the liver, are responsible for coordinating core life activities including fatty acid β-oxidation, tricarboxylic acid (TCA) cycle, and oxidative phosphorylation (OXPHOS). When the organism is exposed to metabolic stressors including a high-fat diet (HFD), this exposure elicits oxidative stress within mitochondria and the ER, thereby promoting hepatic lipogenesis. This process results in dysregulated lipid accumulation and overproduction of reactive oxygen species (ROS), ultimately impairing lipid metabolism and accelerating the progression of MASLD (Deng et al., 2025). Excessive ROS can damage the integrity of mitochondrial membrane lipids and proteins, further deteriorating their function, and forming a vicious cycle (Ipsen et al., 2018; Albillos et al., 2020; Durand et al., 2021; Prasun et al., 2021). Furthermore, MASLD is frequently accompanied by obesity, insulin resistance, and other metabolic anomalies, and is marked by a disequilibrium among hepatic lipid uptake, synthesis, oxidation, and export. The disruption of this homeostasis is not only the direct driver of hepatic lipid accumulation, but also accelerates facilitates the progression of MASLD by inducing mitochondrial dysfunction and related stress responses (Godoy-Matos et al., 2020; Lee et al., 2023).

The mitochondrial quality control (MQC) system serves as the central mechanism for sustaining mitochondrial homeostasis (Vazquez-Calvo et al., 2020). Under physiological conditions, the MQC system complexly controls biogenesis, mitochondrial dynamics (MD), mitochondrial autophagy, protein balance, and other processes crucial for maintaining cellular homeostasis, and sustains cellular energy metabolism homeostasis by regulating mitochondrial quantity, morphology, and function (Vazquez-Calvo et al., 2020). When exposed to stressful environments, mitochondria utilize endogenous antioxidant systems, DNA repair, protein folding, and protein degradation mechanisms to preserve their functional integrity (Ramanathan et al., 2022). Damaged mitochondria can be restored through fusion with healthy mitochondria, while seriously damaged ones undergo fission processes and are eventually eliminated via mitochondrial autophagy (Krishnasamy et al., 2019; Roca-Portoles and Tait, 2021; Ramanathan et al., 2022). Although increasing evidence indicates that MQC dysfunction is closely related to the pathogenesis of MASLD, the precise underlying molecular mechanisms and regulatory networks await full elucidation. Currently, treatment plans for MASLD remain limited. Centered on mitochondria, improving the MQC mechanism may become a potential intervention measure. Therefore, this article aims to elucidate the mechanism of action of MQC in MASLD. Comprehensively analyze the regulatory roles and interrelationships of various components of MQC in MASLD, summarize current advances in MQC-targeting therapeutic strategies, and offer a theoretical foundation and novel perspectives for the precise treatment and drug development of MASLD.

## 2 Mitochondrial quality control mechanism

**Figure 1:**
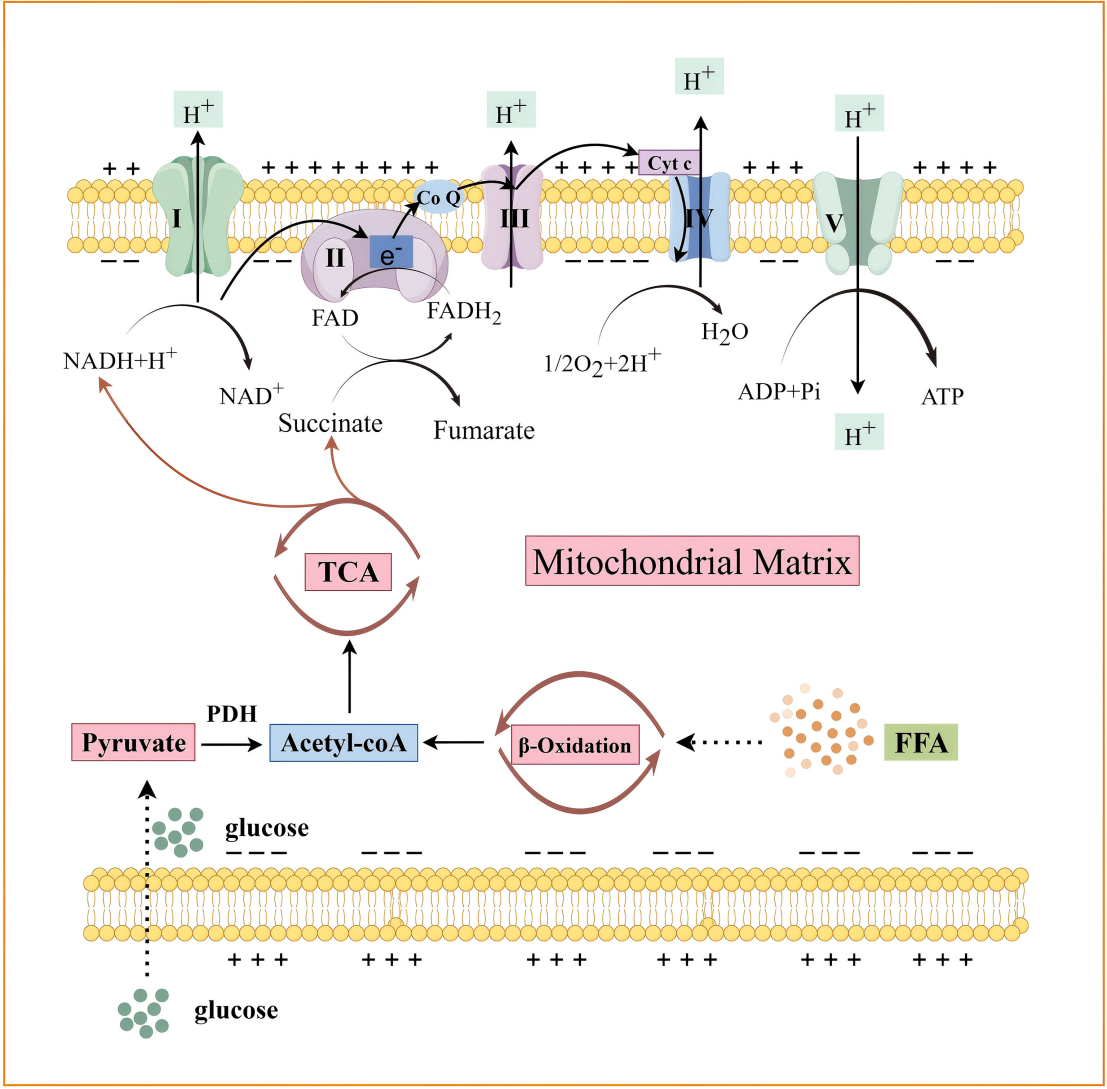
Glucose and fatty acids serve as the primary catabolic substrates, supplying acetyl-CoA for the TCA cycle. Mitochondrial energy production is orchestrated through β-oxidation, the TCA cycle, and ETC-mediated OXPHOS. During β-oxidation, free fatty acids are broken down into acetyl-CoA; meanwhile, glucose is first converted into pyruvate, which is then transformed into acetyl-CoA by the PDH. Acetyl-CoA sustains the TCA cycle and further fuels OXPHOS. Within the TCA cycle, the oxidation of acetyl-CoA generates the high-energy electron carrier NADH, while the oxidation of succinate to fumarate transfers electrons to FADH_2_. NADH and FADH_2_ subsequently donate their electrons to Complex I and Complex II, respectively. As these electron carriers proceed along the ETC, OXPHOS drives the pumping of protons into the intermembrane space, ultimately powering ATP synthase (Complex V) to generate ATP (Perez-Gomez et al., 2020). FFA, free fatty acid; TCA, tricarboxylic acid; NAD^+^, nicotinamide adenine dinucleotide; NADH, reduced nicotinamide adenine dinucleotide; FADH_2_, reduced flavin adenine dinucleotide; FAD, flavin adenine dinucleotide; Co Q, coenzyme Q; OXPHOS, oxidative phosphorylation; Cyt c, cytochrome c; ADP, adenosine diphosphate; ATP, adenosine triphosphate; PDH, pyruvate dehydrogenase. This figure was created by *Figdraw*.

Mitochondria are ubiquitous double-membrane-bound organelles in eukaryotic cells that play a central role in multiple essential biological processes, including energy metabolism, oxidative stress response, ion homeostasis, and signal transduction (Herrmann, 2024). As the cellular “powerhouse” mitochondria not only generate ATP but also act as a central hub in the regulation of metabolic networks. In hepatocytes, mitochondria are highly dynamic organelles indispensable for key metabolic pathways such as lipogenesis, fatty acid β-oxidation and gluconeogenesis, its functional status directly influences hepatic metabolic homeostasis. Mitochondria are structurally organization comprising four distinct compartments: the outer mitochondrial membrane (OMM), the inner mitochondrial membrane (IMM), the mitochondrial intermembrane space and the mitochondrial matrix (Bhatti et al., 2021). The OMM, functioning as the boundary between mitochondria and cytosol, contains multiple mitochondrial outer membrane channels (MOMCs). Equipped with relatively large pores, these MOMCs facilitate the free diffusion of small molecules (e.g., oxygen, water, ions, and proteins with a molecular weight less than 5 kDa) across the OMM (Yang et al., 2024). This high level of permeability allows the ionic environment within the mitochondrial intermembrane space to remain in dynamic equilibrium with the cytosol (Jackson and Theiss, 2020). The IMM folds inward to form cristae, significantly expanding the surface area of the IMM, thereby supplying more reaction room for enzyme and protein complexes on the inner membrane (Yang et al., 2024). The IMM maintains intracellular and extracellular ion balance through controlling ion channel activity, further modulating mitochondrial membrane potential (MMP) and metabolism (Berry et al., 2023). Additionally, contained within the mitochondrial matrix, multiple metabolic enzymes not only directly participate in the metabolism of carbohydrates, lipids, and proteins, but also simultaneously regulate intracellular and extracellular calcium ion homeostasis (Patergnani et al., 2021). The IMM is also the site where mitochondrial OXPHOS occurs. Electron transport chain (ETC) complexes I-V are orderly arranged on the IMM, transferring electrons from NADH to molecular oxygen via the electron transport process (Auger et al., 2015; Nolfi-Donegan et al., 2020; Zhang et al., 2021; Smirnov et al., 2023). This process not only reduces oxygen to water but also establishes a proton gradient across the membrane, which drives ATP synthase (Complex V) to produce ATP, thereby achieving efficient conversion of chemical energy into biologically usable energy (Zhuang et al., 2024). (**Figure 1**)

**Figure 2:**
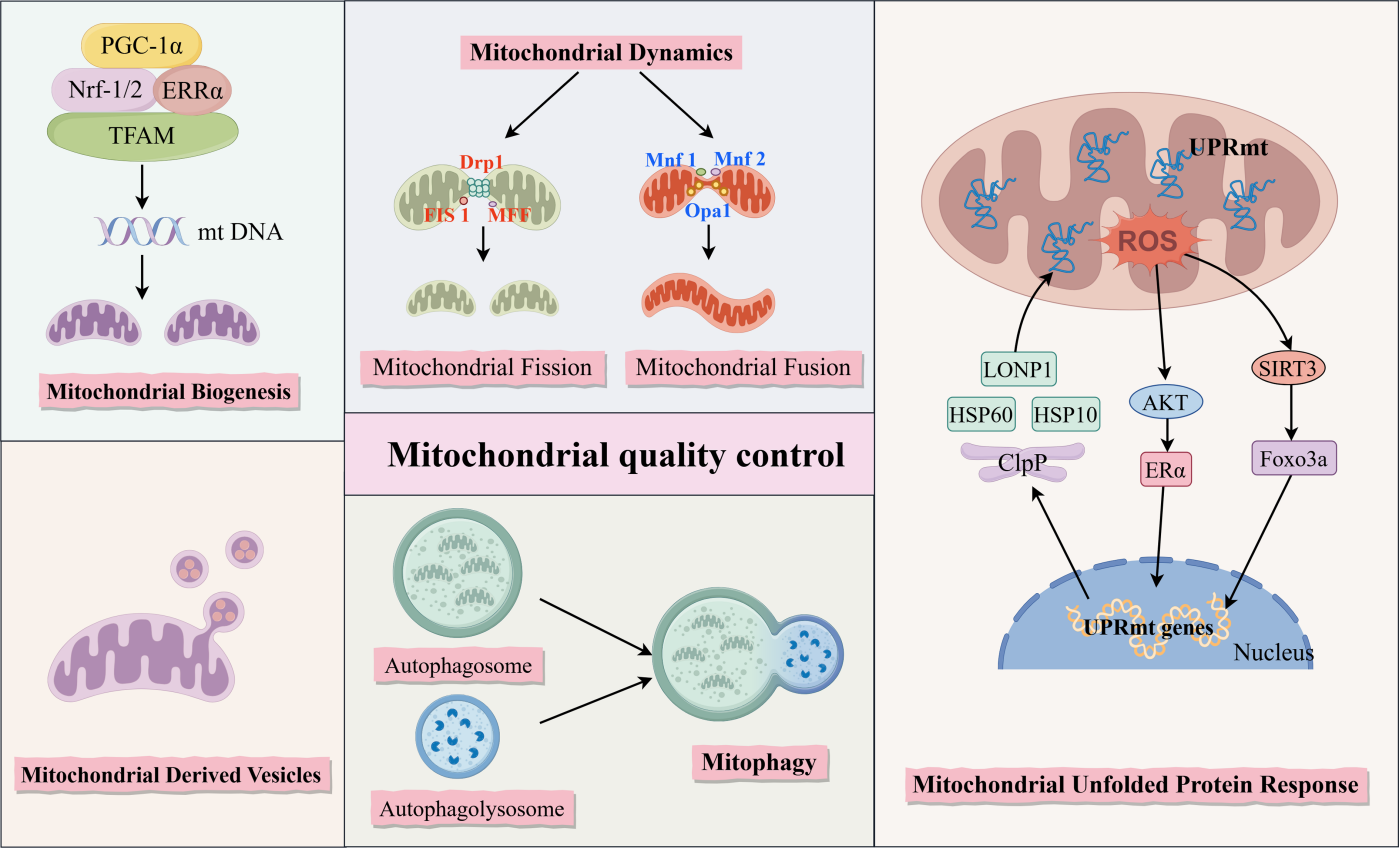
Components of itochondrial quality control (MQC): MQC is a complex process that primarily includes MB, MD (i.e., fission and fusion), UPRmt, mitophagy, and MDVs. PGC-1α, Peroxisome proliferator-activated receptor gamma coactivator 1-alpha; Nrf-1/2, Nuclear respiratory factor 1/2; ERRα, Estrogen-related receptor alpha; TFAM, Mitochondrial transcription factor A; mtDNA, mitochondrial DNA; Drp1, Dynamin-related protein 1; FIS1, Mitochondrial fission 1 protein; MFF, Mitochondrial fission factor; Mfn1/2, Mitofusin 1/2; Opa1, Optic atrophy protein 1; UPRmt, Mitochondrial unfolded protein response; LONP1, Lon protease 1; HSP60, Heat shock protein 60; HSP10, Heat shock protein 10; ClpP, Caseinolytic protease; AKT, Ak strain transforming; ERα, Estrogen receptor alpha; SIRT3, Sirtuin 3; FoxO3a, Forkhead box protein O3a. This figure was created by *Figdraw*.

Recent studies have indicated that some researchers regard mitochondrial dysfunction as a central element in the development of MASLD (Ramanathan et al., 2022). Researches have revealed that adipocytes from obese patients exhibit significant reductions in peroxisome proliferator-activated receptor gamma coactivator 1-alpha (PGC-1α) expression, mitochondrial DNA (mtDNA) content, oxygen consumption rate, and citrate synthase activity. It is also accompanied by markedly impaired activities of mitochondrial respiratory chain complexes. These metabolic abnormalities lead to decreased fatty acid oxidation (FAO) and excessive production of ROS, which results in reduced systemic energy metabolism efficiency and ectopic fat accumulation (Furukawa et al., 2004), thereby disrupting normal metabolic function and promoting obesity-related metabolic diseases such as MASLD (Heinonen et al., 2020). Upon exposure to metabolic stress, cells primarily respond by adjusting mitochondrial activity and metabolic patterns. On one hand, mitochondria fulfill metabolic requirements by means of biogenesis and fusion; on the other hand, they respond to decreased demand via fission and autophagy. Therefore, the MQC system is primarily orchestrated through four dynamic processes: mitochondrial biogenesis (MB), mitochondrial fusion, mitochondrial fission, and autophagy (**Figure 2**). In addition, biological processes including the mitochondrial unfolded protein response (UPRmt) and mitochondrial-derived vesicles (MDVs) also participate in regulating mitochondrial quality. The MQC system requires complex and precise coordination among these components, and disruption in any one of them could result in to mitochondrial dysfunction (Yang et al., 2024).

Mitochondria depend upon the MQC system to maintain their optimal functionality. Experimental models, particularly those employing HFD, have consistently shown that initial mitochondrial impairment disrupts mitochondrial FAO and cellular lipid balance (Yi et al., 2019; Ge et al., 2022; Lam et al., 2025). This disturbance subsequently activates proinflammatory pathways, leading to hepatic steatosis, which exacerbates liver injury and accelerates the progression of MASLD. Impairment of the MQC system directly compromises ATP synthesis and promotes excessive ROS production. This in turn triggers a disruption of cellular energy balance and major metabolic processes, thereby fueling the initiation and progression of hepatic steatosis (Li et al., 2020).

### 2.1 Mitochondrial biogenesis

**Figure 3:**
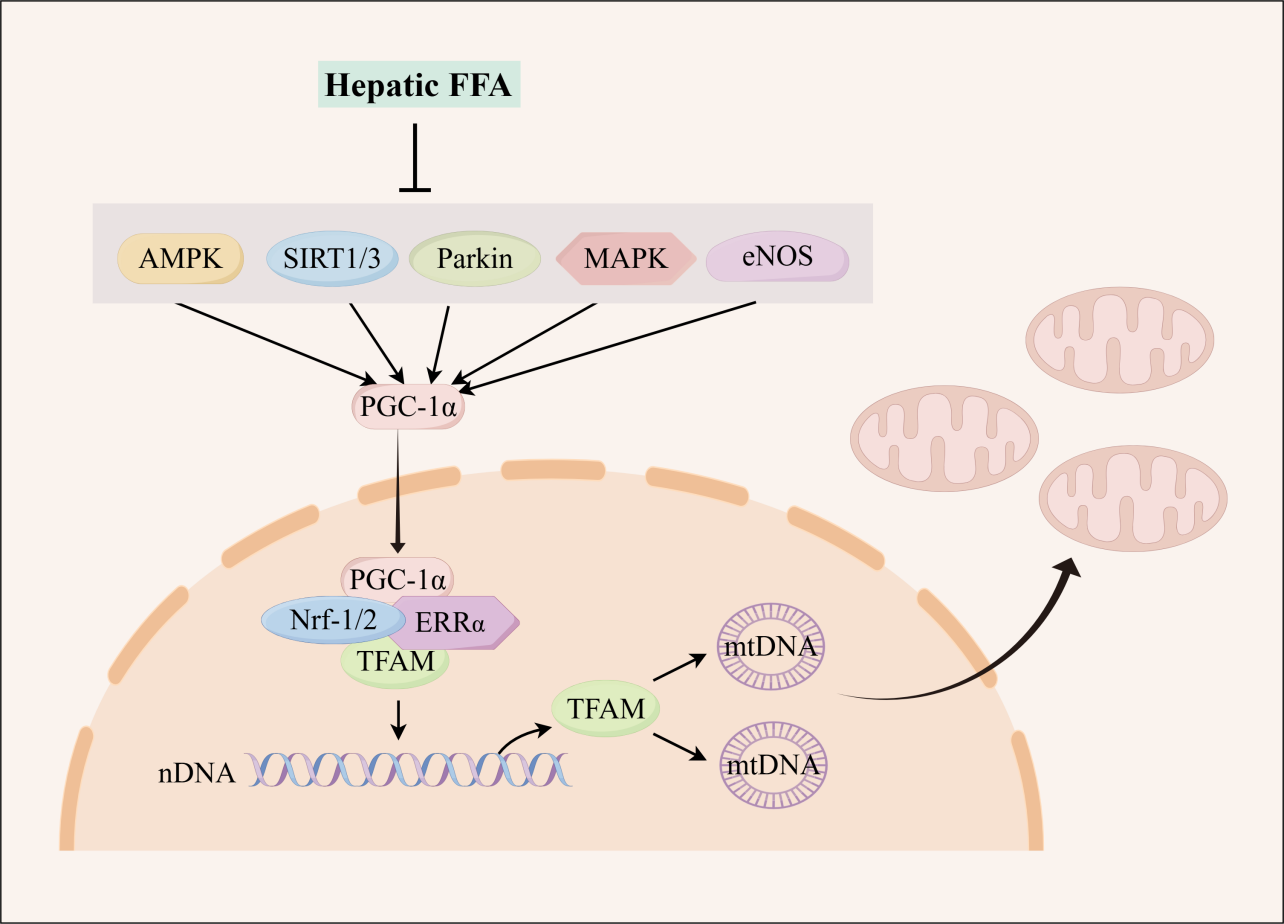
MB constitutes a complex process characterized by multilevel regulation, with PGC-1α acting as its central regulatory molecule. Under physiological conditions, key signaling molecules including Parkin, SIRT1/3, AMPK, eNOS, and MAPK enhance the transcriptional activity and functional capacity of PGC-1α via various regulatory mechanisms, thus facilitating the progression of MB. However, under pathological conditions characterized by excessive fatty acids, the activity of these key signaling nodes is significantly suppressed, leading to a decline in both the transcriptional level and functional PGC-1α activity, ultimately impairing the MB process. AMPK, AMP-activated protein kinase; SIRT1/3, Sirtuin 1/3; Parkin, Parkin RBR E3 ubiquitin protein ligase; MAPK, Mitogen-activated protein kinases; eNOS, Endothelial nitric oxide synthase;PGC-1α, Peroxisome proliferator-activated receptor gamma coactivator 1-alpha; Nrf-1/2, Nuclear respiratory factor 1/2; ERRα, Estrogen-related receptor alpha; TFAM, Mitochondrial transcription factor A; mtDNA, Mitochondrial DNA. This figure was created by *Figdraw*.

MB refers to the intricate biological mechanism through which mitochondria proliferate via self-replication, involving growth and fission to generate new mitochondria (Popov, 2020). As an indispensable component of the MQC system, MB acts as a key adaptable response mechanism for cells to address alterations in metabolic demands and mitochondrial dysfunction. This process not only entails a quantitative increase in mitochondrial mass but, more critically, mediates the generation of fully functional mitochondria to maintain cellular metabolic homeostasis and energy balance (Popov, 2020).Mitochondria are the main site for fatty acid β-oxidation. In MASLD, the mitochondrial biosynthesis regulated by PGC1α is strongly inhibited, resulting in weakened mitochondrial OXPHOS, mitochondrial respiration, and β-oxidation, thereby exacerbating fat accumulation and promoting disease progression (Morio et al., 2021). Notably, PGC-1α is stimulated by the NAD+-dependent deacetylase sirtuin 1 (SIRT1), which subsequently initiates a downstream transcriptional cascade (Koh and Kim, 2021). Following its activation, PGC-1α enters an active state where it orchestrates the expression of mitochondrial transcription factor A (TFAM). It achieves this by cooperating with key transcription factors such as Nrf-1, Nrf-2, and ERRα. The upregulation of TFAM is a critical step that drives mtDNA replication and transcription, thereby promoting mitochondrial biogenesis (MB) (**Figure 3**) (Picca and Lezza, 2015; Piccinin et al., 2019; Tian et al., 2022). Under lipotoxic conditions, excessive fatty acids (FAs) suppress the activity of key regulatory nodes such as AMPK, SIRT1, eNOS, and MAPK, thereby negatively regulating both the transcriptional level and functional activity of PGC-1α, ultimately inhibiting MB (**Figure 3**) (Zhang et al., 2023). Nrf-1 and Nrf-2 are key nuclear transcription factors with critical regulatory functions in mitochondrial biology (Picca and Lezza, 2015). Specifically, they modulate the expression of both subunits of the mitochondrial respiratory complexes and essential mitochondrial transcription factors. Among the latter, TFAM, TFB1M, and TFB2M are prominent targets of their regulation (Picca and Lezza, 2015). TFAM, as a critical regulator of mtDNA copy number (mtDNA-CN) in mammals, binds to mtDNA at multiple sites and is mainly responsible for maintaining mtDNA packaging and initiating transcription, serving as an important bridge linking nuclear genomic regulation with mitochondrial genomic expression (Picca and Lezza, 2015). The nuclear-encoded and mitochondrial-encoded proteins produced through transcription and translation are subsequently assembled into nascent mitochondria, thereby maintaining mitochondrial morphology and functional homeostasis.Under physiological conditions, cellular responses to energy demands lead to upregulation or downregulation of PGC-1α and its downstream transcriptional regulators (such as Nrf-1, Nrf-2, and ERRα) to stimulate or inhibit MB (Popov, 2020). During the early compensatory phase of MASLD, MB may be transiently activated as a protective mechanism, resulting in upregulated expression of PGC-1α, PGC-1β, and their downstream targets (Dornas and Schuppan, 2020). However, with disease progression, persistent lipotoxicity ultimately leads to suppression of the PGC-1α signaling pathway. In advanced MASLD, a set of interconnected mitochondrial alterations emerges, characterized primarily by increased mitophagy, reduced mitochondrial mass, decreased mtDNA-CN, and diminished PGC-1α expression. This pathological state is further compounded by impaired ATP generation. Collectively, these deficits drive and sustain a vicious cycle of progressive hepatic mitochondrial depletion and dysfunction (Lee et al., 2018).

Furthermore, the expression levels of SIRT1 and its downstream effector PGC-1α were markedly decreased in both *in vivo* MASLD mouse models and *in vitro* cell models (Jiang et al., 2021). The decreased expression of PGC-1α leads to increased lipid accumulation and ROS generation, which subsequently trigger reduced cell viability and apoptosis (Jiang et al., 2021). In this context, SIRT1 plays a dual fundamental role. Not only does it promotes the transcription of genes essential for energy and metabolic homeostasis in MB, but also ensures their normal functioning by mediating the deacetylation of downstream target proteins (Yang et al., 2021). As a key mitochondrial NAD+-dependent deacetylase, SIRT3 ensures mitochondrial integrity by modulating ETC efficiency, safeguarding mtDNA, and stimulating MB (Ahmedy et al., 2022; Akter et al., 2023; Jin et al., 2023). Liu et al. delineated that SIRT3 protects hepatocytes from oxidative damage and preserves mitochondrial morphological characteristics and structural intactness by enhancing antioxidant capacity, improving MB, and preventing excessive mitochondrial fragmentation (Liu et al., 2017). Studies have established that PGC-1α not only mitigates hepatic inflammatory responses and hepatocyte apoptosis but also modulates MB by activating downstream targets such as Nrf-1. In addition, reduced PGC-1α expression disrupts the activity of Nrf-1 and TFAM, further intensifying mitochondrial dysfunction and impairing cellular energy metabolism (Léveillé et al., 2020; Abu Shelbayeh et al., 2023; Wang W. et al., 2024). Experimental studies have shown that in the liver of mice with NAFLD induced by a choline-deficient, ethionine-supplemented diet, exhibit suppressed PGC-1α expression, impaired MB, and downregulated TFAM expression (Aharoni-Simon et al., 2011). Furthermore, in a mouse model of MASLD induced by 23 weeks of HFD feeding, SIRT1-mediated MB was significantly suppressed, accompanied by a consistent downregulation of SIRT1, Nrf-1 and TFAM protein expression. These findings were also reproduced in the AML12 cell model (Yang et al., 2023). A comparative study has shown that, compared with a healthy control group, patients with MASLD exhibit significantly impaired mitochondrial fatty acid oxidation capacity, regardless of whether they are in the early stage of simple steatosis or have progressed to steatohepatitis (MASH). Furthermore, the expression levels of markers associated with mitochondrial dynamics (including biosynthesis, autophagy, fission and fusion) are generally downregulated (Moore et al., 2022). It is worth noting that patients with MASLD exhibit ultrastructural changes consistent with giant mitochondria, characterised by a significant reduction in the surface-to-volume ratio and disruption of the IMM and cristae, suggesting mitochondrial dysfunction (Shami et al., 2021).

Studies show that mitochondria store their genetic material in closely arranged circular DNA molecules (known as mtDNA) (Panel et al., 2018). As compared with genomic DNA, mtDNA exhibits lower stability owing to the lack of TFAM packaging and is susceptible to enzymatic degradation and oxidation (Rantanen and Larsson, 2000; Torregrosa-Muñumer et al., 2015; Wu et al., 2021). Furthermore, mtDNA lacks histone-mediated protection and efficient DNA repair machinery, thereby contributing to a high mutation rate and increased susceptibility to oxidative damage induced by ROS. Accumulation of such mtDNA lesions ultimately impairs hepatic lipid metabolic homeostasis in hepatocytes (Wu et al., 2021). Following an imbalance in mtDNA repair and damage, mtDNA fragments are released. Once released, they may either accumulate within the cytoplasm or be secreted outside the cell (Fang et al., 2021). Damaged mtDNA induces ETC defects and suppresses MB. Moreover, upregulated ROS levels modulate insulin and innate immune signaling, affect the expression of lipid metabolic enzymes, and lead to hepatic lipid metabolic dysregulation, thereby promoting MASLD progression (Chen et al., 2020). A decrease in mitochondrial copy number typically indicates reduced mitochondrial gene expression capacity (Mishmar et al., 2019). Therefore, assessment of mtDNA-CN, the mtDNA-to-nuclear DNA (nDNA) ratio, and mitochondrial gene expression levels are commonly used as reliable markers for evaluating the extent of MB (Andres et al., 2017). When the mtDNA: nDNA ratio increases along with elevated mitochondrial gene expression levels, the number of newly formed mitochondria correspondingly rises (Andres et al., 2017). MtDNA-CN represents the abundance of mtDNA relative to nDNA within cells and is associated with mitochondrial enzyme activity and ATP production. The lack of a comprehensive DNA repair mechanism is a key factor rendering mtDNA more vulnerable to damage than nDNA (Nadalutti et al., 2022). Furthermore, the results of liver biopsy tissue analysis indicate that the number of mtDNA CN in the livers of patients with MASLD is significantly lower than in healthy controls (Sookoian et al., 2010; Pirola et al., 2015). MtDNA levels are also significantly reduced in liver tissue from patients with cirrhosis and in HCC tissue (Tang et al., 2017). In the MASH animal model induced by MCD and HFD diets, giant mitochondria were observed in the liver in both groups. Compared with the HFD group, the number of mtDNA CN in the liver was significantly reduced in the MCD group; this phenomenon was closely associated with the significant upregulation of gene expression levels related to MB and degradation in the MCD model (Arao et al., 2020).

As a key mechanism for maintaining metabolic homeostasis in hepatocytes, MB exerts a dual role in the pathogenic mechanism of MASLD. In the initial phases of the disease, a key compensatory response is the activation of MB. This response works to offset metabolic stress through the generation of new, functional mitochondria. However, with disease progression, persistent oxidative stress, inflammatory responses, and metabolic dysregulation culminate in MB process being inhibited. A deeper elucidation of the regulatory mechanisms controlling MB and its role in MASLD is pivotal for advancing our understanding of the disease’s pathogenesis. This knowledge, in turn, is essential for developing targeted therapeutic strategies aimed at mitochondria.

### 2.2 Mitochondrial dynamics

MD refers to the regulatory mechanisms that maintain the shape, structure, number and functional balance of mitochondria through the coordinated process of fusion and fission (Zhang H. et al., 2020). Under physiological conditions, mitochondrial fission is largely driven by dynamin-related protein 1 (Drp1), and mitochondrial fusion is largely directed by mitofusins 1 and 2 (Mfn1/2), which promote outer membrane fusion, and the protein optic atrophy protein 1(Opa1), which controls inner membrane fusion (Wu et al., 2025). Mitochondria maintain the dynamic equilibrium of the IMM and OMM through the coordinated processes of mitochondrial fusion and fission, ensuring efficient ATP production, proper fatty acid β-oxidation, and appropriate control of ROS generation (Wu et al., 2025).

Research reports that mitochondrial fission includes two different fission ways. Peripheral mitochondrial fission facilitates the mitophagy mediated mitochondrial degradation, whereas intermediate zone mitochondrial fission is required for maintaining MB and MD (Kleele et al., 2021). Mitochondrial fission refers to the process by which the OMM fission protein 1 (Fis1) and mitochondrial fission factor (MFF) recruit Drp1 at the OMM (Li P. et al., 2019; Lee et al., 2022; Song et al., 2024). This recruitment induces the translocation of Drp1 from the cytosol to the OMM, where Drp1 forms a helical structure, constricts, and splits the mitochondrion, separating a single mitochondrion into two smaller fragments. The removal of functionally impaired mitochondria through mitophagy facilitates the decrease of damaged/dysfunctional mitochondria. The fusion of mitochondria involves the incorporation of the mitochondrial fusion protein 2 (Mfn2) into the OMM of two mitochondria, regulating their fusion into a single organelle. This process protects the stability of mtDNA and reduces structural and functional defects (Chan, 2020; Huang et al., 2021; Sidarala et al., 2022). Mfn2 is localized on both the OMM and the ER membrane. On the OMM, it links adjacent mitochondria. On the ER surface, Mfn2 forms dimers either with Mfn2 or with Mfn1 on mitochondria. This dimerization tethers the ER to mitochondria, a connection that facilitates mitochondrial Ca2+ transport (de Brito and Scorrano, 2008). In terms of mitochondrial characteristics, the division of mitochondria maintains the balance of their components (including DNA, proteins and metabolites) (Anzell et al., 2018). Fusion and fission produce a spectrum of mitochondrial morphologies, including spherical, tubular, and networked forms. This diversity in shape supports adaptation to different physiological conditions (Lurette et al., 2022). External stimuli like metabolic, oxidative, or inflammatory stress can lead to disrupted mitochondrial dynamic equilibrium. This is frequently driven by aberrant expression of regulatory proteins, such as the fusion proteins Mfn1/2 and the fission protein Drp1 (Portincasa et al., 2024). This phenomenon is characterized by enhanced fission and suppressed fusion, leading to fragmented mitochondrial networks and consequent losses in both structural integrity and functional stability (Portincasa et al., 2024).

**Figure 4:**
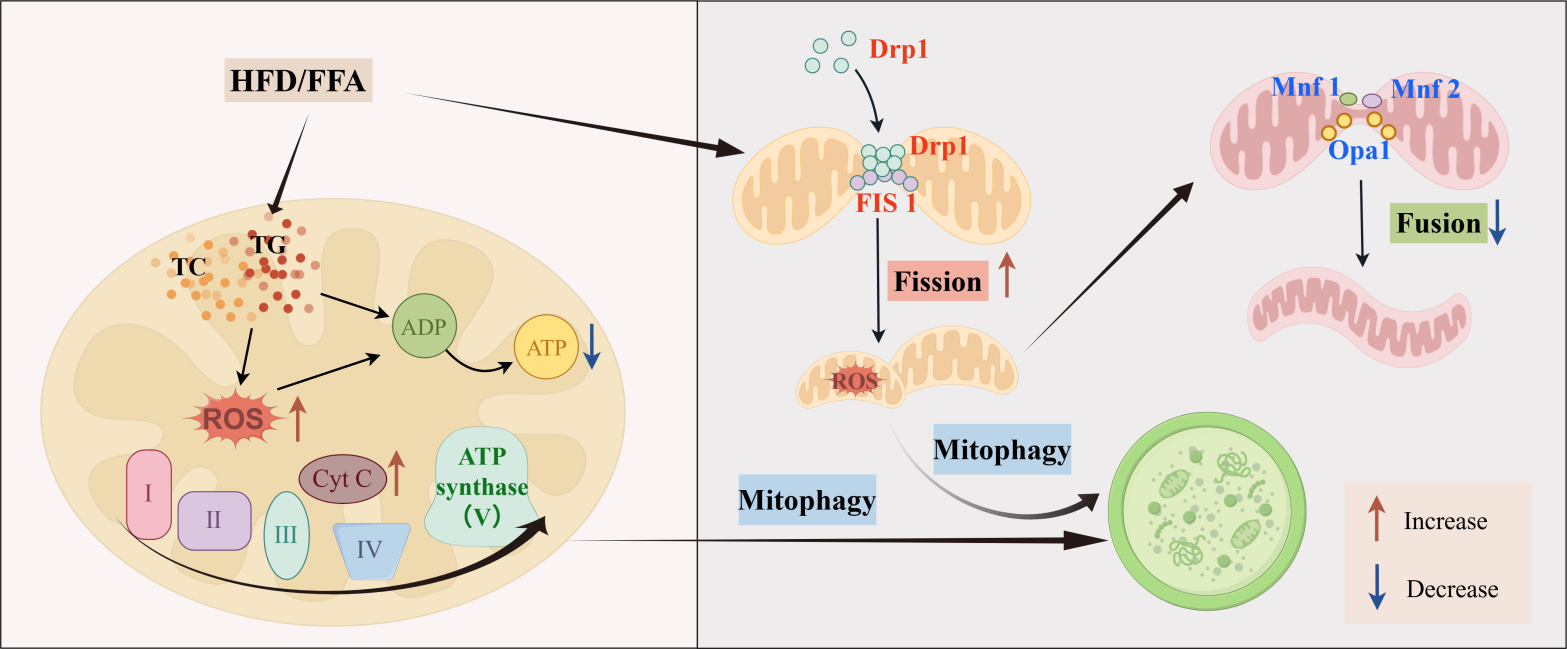
HFD disrupts MD, affecting MQC. This is primarily characterized by enhanced mitochondrial fission and suppressed fusion. Following fission, the resulting mitochondria face distinct fates: some undergo clearance by autophagic lysosomes, while others may be recycled through re-fusion. HFD promotes intracellular lipid accumulation, which impairs mitochondrial function. This impairment is reflected in increased production of ROS and Cyt C, coupled with inhibited ATP synthesis. These changes exacerbate mitochondrial dysfunction. Together, damaged mitochondria are eliminated via the mitophagy pathway. TG, triglyceride; TC, total cholesterol; ADP, adenosine diphosphate; ATP, adenosine triphosphate; ROS, Reactive oxygen species; Cyt C, Cytochrome C; Drp1, Dynamin-related protein 1; FIS1, Mitochondrial fission 1 protein; Mfn1/2, Mitofusin 1/2; Opa1, Optic atrophy protein 1. This figure was created by *Figdraw*.

Evidence indicates that mitochondrial fusion and fission are dysregulated in obesity and MASLD. This is driven by excessive fatty acid loading, which upregulates fission-related gene expression, manifests as increased mitochondrial fission and decreased fusion (Galloway et al., 2014; Wai and Langer, 2016; Zhang et al., 2023). Furthermore, enhanced mitochondrial fission in hepatocytes correlates with the early stages of MASLD. Inhibition of this process can alleviate hepatic inflammation and fibrosis, as verified in mouse models (Miyao et al., 2022; Elbadawy et al., 2023; Quan et al., 2023). *In vitro* exposure to FA (such as oleic acid and palmitic acid) results in hepatocyte steatosis, increased mitochondrial fission, decreased MMP, cytochrome C (Cyt C) release, and elevated ROS (Zhang et al., 2019) (**Figure 4**). Research in both animal and cellular models points to a disrupted balance in mitochondrial fission and fusion (Legaki et al., 2022). This dysregulation triggers mitochondrial dysfunction, manifesting as decreased ATP output and increased oxidative stress. These changes thereby promote to the progression of lipid accumulation and inflammatory responses (Legaki et al., 2022). Compared with the HFD model, mRNA levels of key regulators of mitochondrial biogenesis (SIRT1 and TFAM), fusion (Mfn1 and Mfn2) and fission (Drp1) were significantly reduced in the MCD diet model. Similarly, compared with HFD mice, mRNA expression of key mitochondrial autophagy regulators (namely BNIP3, Fundc1 and PINK1) was significantly downregulated in MCD mice. In the high-cholesterol diet model, exactly the same results were observed as in the HFD model (Alarabi et al., 2026).Mitochondria are involved in a diverse array of cellular functions beyond energy production. These include: the maintenance of homeostasis (such as calcium and protein balance), biosynthesis of heme and lipids, signal modulation through ROS generation and the regulation of programmed cell death (apoptosis) (Liu et al., 2023). These pathological morphological changes impair fundamental mitochondrial bioenergetic functions. This disrupt MQC mechanisms results in the accumulation of dysfunctional mitochondria within hepatocytes, which in turn promotes the development and progression of MASLD. Understanding the regulation of MD in MASLD is therefore key to both unraveling disease mechanisms and discovering new therapeutic avenues.

### 2.3 Mitochondrial unfolded protein reaction

Beyond the typical mitophagy pathways, mitochondria possess proteases that recognize misfolded or oxidized mitochondrial, promoting mitochondrial degradation, a process known as the UPRmt (Ma et al., 2024). The accumulation of misfolded or unfolded proteins within the mitochondrial matrix triggers a retrograde signaling response to the cell nucleus, increasing the production of proteases and protein folding chaperones (Shpilka and Haynes, 2018; Torres et al., 2024; Zhang MY. et al., 2025). UPRmt is a key mechanism within the MQC system that is both triggered by mitochondrial dysfunction and functions to restore mitochondrial function and alleviate cellular stress (Suárez-Rivero et al., 2022a; Vo et al., 2022; Longhitano et al., 2024; Yang M. et al., 2025). The UPRmt is a communication pathway that relies on signals from both the nuclear and mitochondrial genomes. When coordination between these genomes is disrupted, it leads to nucleocytoplasmic imbalance, affecting protein expression (Wu et al., 2024). Notably, mitochondria comprise both nuclear-encoded and mitochondrial-encoded proteins. These proteins assemble within the organelle to form functional protein complexes, which are localized in the mitochondrial matrix and IMM (Harrington et al., 2023). In response to the substantial accumulation of misfolded proteins in the matrix, the SIRT3-mediated UPRmt is activated. This process promotes the expression of mitochondrial antioxidant genes while simultaneously eliminating irreversibly damaged mitochondria via mitochondrial autophagy (Pareek and Pallanck, 2018). Studies have identified that UPRmt activation is triggered by two key signaling molecules released into the cytoplasm: mitochondrial ROS released into the cytoplasm and mitochondrial protein precursors (c-mtProt) accumulated in the cytoplasm. Specifically, proteomics and genetic approaches have revealed that mitochondrial misfolding stress (MMS) leads to mitochondrial ROS release into the cytoplasm on one hand; simultaneously, MMS causes mitochondrial protein import defects, resulting in c-mtProt accumulation in the cytoplasm (Sutandy et al., 2023).

In clinical samples taken from patients with MASLD and in animal models, increased expression of UPRmt-related genes and proteins has been observed. These findings confirm a link between the progression of MASLD and the activation of the UPRmt (Urbina-Varela et al., 2020). This suggests that activating UPRmt may be a corrective strategy to restore mitochondrial protein balance, thereby reducing cellular stress in MASLD (Suárez-Rivero et al., 2022b). However, some studies indicate that ongoing or severe metabolic stress (e.g., ER stress and oxidative stress) of MASLD, which can attenuate the UPRmt response, thereby exacerbating liver damage (Ajoolabady et al., 2023). Yuan et al. established a MASLD mouse progression model using the Amylin Liver NASH diet. After 14 weeks of Amylin Liver NASH diet intervention, mice showed significantly increased hepatic lipid deposition; meanwhile, the gene and protein expression of UPRmt marker molecules including heat shock protein 70 (HSP70), heat shock protein 60 (HSP60), caseinolytic protease (ClpP), and Lon peptidase 1 (LONP1) were markedly upregulated, and UPRmt was obviously activated. After 18 weeks of Amylin Liver NASH diet intervention, liver lipid deposition further worsened, the expression of UPRmt-related proteins decreased significantly, UPRmt function was impaired and failed to exert compensatory effects, and the lesions progressed to advanced MASLD and further developed into MASH (Yuan et al., 2025).The specific mechanism is as follows: In cases of hepatic steatosis, the accumulation of unfolded or misfolded mitochondrial proteins within the mitochondrial matrix triggers the UPRmt. The UPRmt can alleviate cellular stress arising from impaired mitochondrial function (Suárez-Rivero et al., 2022a). Large lipid droplets forming within hepatocytes further exacerbate excessive inflammation, ER stress, and oxidative stress. Among these, ER stress triggers the UPR, which indirectly promotes hepatocyte inflammation and inflammasome activation through its downstream signaling pathways (Ding et al., 2023). Drive the evolution of simple fatty liver to MASH (Longhitano et al., 2024).

Accumulating evidence demonstrates that UPRmt confers protective effects in liver metabolic diseases (Haynes et al., 2007; Zhu et al., 2021; Yuan et al., 2025). However, under continued high glucose and high lipid stress, mitochondria show pronounced damage. At this phase, mitochondrial and mitochondrial cristae numbers decrease, which suggests disrupted fusion-fission balance (i.e., increased fission and decreased fusion) (Wu et al., 2019). Under such persistent and excessive stress conditions, the clearance capacity of UPRmt may reach its limit: unfolded and misfolded proteins cannot be effectively cleared by UPRmt and accumulate in mitochondria. This accumulation, exacerbates increased mitochondrial fission and decreased fusion (Wu et al., 2019). Separately, protein misfolding initiates a detrimental cascade. It begins with reduced ATP synthesis and a surge in superoxide anions. These conditions then promote an increase in pro-apoptotic molecules, ultimately leading to cell death (Cai et al., 2022). Despite these challenges, UPRmt, as the core defense mechanism against mitochondrial damage, achieves its protective functions primarily through the following pathways: refolding and repair of unfolded and misfolded proteins; selective degradation of damaged proteins or entire mitochondria through mitophagy; regulation of MB to replenish healthy mitochondria; and amelioration of oxidative stress (Deng and Haynes, 2017). Through these synergistic actions, UPRmt strives to rebuild a stable mitochondrial environment (Münch, 2018). In conclusion, UPRmt is a highly preserved stress response mechanism that keeps mitochondrial protein homeostasis, with its core role in safeguarding mitochondrial function, thus playing an indispensable role in the survival and health maintenance of most organisms.

### 2.4 Mitophagy

The whole degradation process of non selective transport of metabolic wastes (nucleic acids, proteins and organelles) to lysosomes is called autophagy (Nakatogawa, 2020). In addition, it also acts as a selective autophagy system to regulate the clearance of specific organelles. Biological process of autophagy: double membrane autophagosomes phagocytize cellular components, form autophagosomes with lysosomes, and degrade them through hydrolases in lysosomes to maintain cell homeostasis (Garza-Lombó et al., 2020). Autophagy primarily consists of three types: macroautophagy, microautophagy, and chaperone-mediated autophagy (Yu et al., 2018). Mitophagy, ER-phagy, and lipophagy are forms of selective autophagy (Pang et al., 2018). Among these, mitophagy has emerged as a research hotspot in liver diseases; however, the mechanisms underlying mitophagy in MASLD remain unclear and warrant further investigation.

The biogenesis of mitophagosomes involves the formation, extension, maturation, and closure of isolation membranes (IM), followed by fusion with lysosomes to form autophagosomes. Under stress or injury, mitophagy averts the accumulation of damaged mitochondria and increased ROS homeostatic levels, thereby avoiding oxidative stress and cell death (Garza-Lombó et al., 2020). Scientific evidence indicates that oxidative stress can activate mitophagy. In this process, dysfunctional or superfluous mitochondria are selectively encapsulated into autophagosomes. These autophagosomes are subsequently delivered to lysosomes for degradation. This clearance mechanism helps to regulate mitochondrial quantity and support stable cellular energy metabolism (Onishi et al., 2021; Gu et al., 2024; Xia et al., 2025). Damaged mitochondria are selectively eliminated through autophagy or direct lysosome-mediated micromitophagy. Furthermore, mitophagy promotes cell survival by eliminating damaged mitochondria, as cytosolic release of Cyt C during mitochondrial injure correlates with apoptosis-induced programmed cell death (Wanderoy et al., 2020).

The initiation of mitophagy depends on precise molecular recognition mechanisms. Research has revealed that MMP serves as the primary signal for inhibiting or initiating mitophagy (Swerdlow and Wilkins, 2020). Bioenergetic reactions generated in the mitochondrial respiratory chain maintain the mitochondrial electrochemical gradient, or MMP. When MMP decreases or function becomes aberrant, PTEN-induced kinase 1 (PINK1) becomes active and accumulates on the OMM, recruiting parkin RBR E3 ubiquitin protein ligase (Parkin) and ubiquitin through phosphorylation (Lin et al., 2020). Activated Parkin ubiquitinates OMM proteins, and these ubiquitinated mitochondria are recognized and bound by autophagy receptor proteins, subsequently binding to microtubule-associated protein light chain 3 (LC3) on the autophagosome membrane to form mitophagosomes (Yoo and Jung, 2018). Ultimately, mitophagosomes are completely degraded by lysosomal hydrolases. In summary, damaged and dysfunctional mitochondria can be recognized and degraded through autophagy receptor proteins such as PINK1 and Parkin, maintaining mitochondrial quality (Lin et al., 2020).

Accumulating evidence indicates that mitophagy defects impede effective clearance of damaged mitochondria, thereby exacerbating hepatocyte injury. Similarly, deletion of the mitophagy regulator BNIP3 induces lipid synthesis while decreasing hepatic fatty acid β-oxidation levels in mice. Supporting evidence comes from studies in obesity-induced hepatic steatosis mouse models. These models exhibit dramatically reduced levels of hepatic mitophagy. This observation points to a potential link between impaired hepatic mitophagy and the development of MASLD (Sun et al., 2015). In the livers of NAFLD rats induced by high-fat diet, the expressions of autophagy-related proteins such as LC3-II/I, ATG7 (Autophagy-related protein 7), PINK1, and Parkin significantly decreased, while the expression of SQSTM1/p62 protein significantly increased, and mitochondrial autophagy was inhibited (Yao et al., 2023). Macrophage Stimulating 1 (Mst1) is a novel upstream regulator of mitochondrial autophagy. In the MASLD mouse model induced by HFD, the expression of Mst1 in the liver is often upregulated. Mst1 down-regulates Parkin expression by inhibiting the AMPK pathway, thereby suppressing the Parkin-related mitochondrial autophagy activity. After mitochondrial autophagy is inhibited, damaged mitochondria cannot be promptly cleared, leading to mitochondrial homeostasis imbalance, decreased mitochondrial membrane potential, excessive production of mitochondrial ROS, and increased opening of mPTP. At the same time, it induces oxidative stress disorder in the liver, massive release of inflammatory factors, and initiates caspase-9/3-mediated mitochondrial apoptosis in liver cells, aggravating hepatic steatosis, liver tissue damage and fibrosis, and thereby promoting the occurrence and development of NAFLD (Zhou et al., 2019). After 14 weeks of HFD feeding, rats developed pronounced lipid deposition and hepatic steatosis (Cioffi et al., 2022). This process led to a marked decline in hepatic mitochondrial quality. The deterioration was evident across several parts, such as increased mitochondrial ROS production, aggravated mtDNA damage, reduced mitochondrial fusion, and impaired mitophagy. Excessive lipid accumulation can have dual detrimental effects: it may both suppress mitophagy and exacerbate MASLD-related inflammatory responses. Conversely, interventions that reduce lipid overload can help reinstate mitochondrial autophagic function, which in turn mitigates liver disease (Ma et al., 2020).

### 2.5 Mitochondria-derived vesicles

Mitochondria-derived vesicles (MDVs) represent an alternative pathway that complements mitophagy. They are small vesicles generated through the outward budding of the mitochondrial membrane. They maintain MQC by transporting their contents to lysosomes for degradation or releasing them into the extracellular space, thereby ensuring normal cellular function (König et al., 2021).

Selective loading of mitochondrial components can be performed, including those from the outer membrane (e.g., voltage-dependent anion channel, translocase of the outer membrane, monoamine oxidases), the inner membrane (e.g., mitochondrial calcium uniporter protein, translocase of inner mitochondrial membrane 23, ETC complex II/III/IV, Opa1), as well as the matrix (e.g., pyruvate dehydrogenase, tricarboxylic acid cycle enzymes, molecular chaperones, mitochondrial ribosomal proteins). There are significant differences in the cargoes carried by MDVs in different species and cell types; some MDV subgroups can carry mtDNA, and the RNA loading characteristics of this are currently unclear. In addition, MDVs can also recruit cytoplasmic and other organelle adapter proteins to participate in their own targeted transport process (Hazan Ben-Menachem et al., 2024). This process enables the timely clearance of damaged mitochondrial components. This direct action supports two key outcomes: it helps restore intracellular homeostasis and maintains mitochondrial structural and functional integrity (Popov, 2022). MDVs degrade mitochondria that have not undergone depolarisation, providing a rapid pathway for MQC regulation. This process selectively eliminates defective mitochondrial proteins and lipids (McLelland et al., 2014).

MDVs act as an early-acting cellular defense mechanism promptly activated upon recognizing mitochondrial damage. MDVs can be cleared via several distinct pathways. These include: (1) transport to lysosomes for degradation (Soubannier et al., 2012); (2) delivery to peroxisomes; (3) packaging into extracellular vesicles (EVs) for secretion. This diversity in routing underscores the complexity of MQC (Peng et al., 2025). A key pathway within MQC involves the PINK1/Parkin system. This system tags and packages damaged mitochondrial components into MDVs. These vesicles are then transported to lysosomes for degradation, a clearance process that helps preserve mitochondrial homeostasis (McLelland et al., 2014).

In the liver, acute metabolic stress induced by a HFD and lipotoxic oxidative stress trigger a compensatory increase in MDV biogenesis. This serves as an early adaptive response to mitochondrial damage and precedes the onset of large-scale mitophagy (König et al., 2021). When LC3-mediated autophagosome formation is impaired, MDVs can serve as a compensatory quality control pathway. This pathway involves the selective transport of cargo like misfolded proteins, oxidized lipids, or repaired mtDNA into specialized MDVs. These compartments are subsequently delivered to either lysosomes or peroxisomes for degradation (Sugiura et al., 2014; Towers et al., 2021; Wilson et al., 2024). Recent studies have further revealed novel functions of EVs in MQC. EVs not only transmit molecules such as mtDNA and protein complexes of the respiratory chain, but can also transfer entire organelles, including damaged mitochondria. Importantly, under conditions of lysosomal dysfunction, the elimination of damaged mitochondria through mitochondrial EVs is correspondingly enhanced (Sanz-Ros et al., 2023; Sharma, 2023; Kong et al., 2025). In addition, some MDVs also target other organelles, particularly peroxisomes, and participate in lipid metabolism, and redox homeostasis (Rapushi et al., 2026). Mild oxidative stress can trigger the biogenesis of MDVs, which encapsulate oxidatively damaged mitochondrial proteins and lipids and deliver them to lysosomes for degradation, thereby alleviating oxidative stress and restoring cellular homeostasis. When MDV-mediated protective mechanisms fail, sustained accumulation of ROS and lipotoxicity drive the progression from simple hepatic steatosis to steatohepatitis and fibrosis (König and McBride, 2024). In recent years, MDV release has also been recognized as a biomarker for liver diseases (Thietart and Rautou, 2020). MDVs serve as a crucial component of MQC and participate in the maintenance of mitochondrial homeostasis (Roque et al., 2020). At present, the specific mechanisms by which MDVs are involved in the occurrence and progression of MASLD remain unclear and require further investigation. Therefore, in-depth elucidation of the regulatory role of MDVs in the progression of MASLD possesses important scientific research significance and potential clinical value.

## 3 Targeting mitochondrial regulation as a potential therapeutic approach for MASLD

The global incidence of chronic metabolic diseases continues to rise. This trend is driven in part by widespread shifts toward high-calorie, high-protein diets, reflecting changes in lifestyle and dietary patterns. Mitochondria are central to cellular energy metabolism and are crucial for maintaining systemic metabolic balance. Research indicates that when mitochondria become dysfunctional, they fail to sustain energy homeostasis. This failure promotes lipid accumulation in hepatocytes and disrupts metabolic processes, key drivers in the onset and progression of MASLD (Wang SW. et al., 2020). Given this central role, mitochondria represent a logical therapeutic target for MASLD. Consequently, a major focus of current research is the development of strategies that can translate into effective clinical interventions.

### 3.1 A strategy for directly targeting mitochondrial quality control in the treatment of MASLD

Mitochondria sustain organismal health through their role in energy metabolism. In MASLD, mitochondrial dysfunction coupled with MQC failure acts as a key driver of disease pathogenesis. The MQC system functions as an intracellular surveillance and degradation system. It does this by identifying and eliminating dysfunctional or damaged mitochondria. This process is essential for preserving the structural and functional integrity of the mitochondrial network. Therefore, therapeutic strategies for MASLD must initially target mitochondrial function. A complementary and necessary approach involves pharmacological interventions designed to restore core MQC processes, such as mitophagy, MB, fusion, and fission. This chapter focuses on current pharmacological strategies designed to enhance mitochondrial function and optimize the MQC system, ultimately seeking to provide new perspectives for mitigating or reversing MASLD progression.

#### 3.1.1 Promote mitochondrial biogenesis

MB is a key process for hepatocyte protection and a potential target in MASLD treatment. Nevertheless, the precise mechanisms by which it confers these benefits remain to be fully elucidated. Consequently, promoting MB stands as a major strategic direction in the development of MASLD therapies. MB agonists work by activating specific signaling pathways that upregulate key regulatory factors (PGC-1α, Nrf-1, and Nrf-2). This upregulation subsequently drives multiple beneficial outcomes: increased mitochondrial biogenesis, improved hepatic function, reduced lipid accumulation, and delayed MASLD progression (Wang SW. et al., 2020).

PGC-1α coordinates MB and cellular energy metabolism through its governance of gene expression, particularly targeting those involved in FAO and MB itself (Lee J. et al., 2019). MQC is maintained through a dynamic balance between biogenesis and clearance. A key regulator of this process is AMP-activated protein kinase (AMPK). This kinase promotes mitophagy by activating the PINK1/Parkin pathway, which leads to the selective removal of damaged organelles and a reduction in lipid peroxidation injury (Xue et al., 2024). This process cooperates with PGC-1α-mediated MB. Together, they establish a complete MQC cycle, with the core outcome of maintaining the long-term stability of fatty acid β-oxidation function. As a central regulator of antioxidant responses (Lin et al., 2025), Nrf-2 provides a major line of defense against mitochondrial ROS by inducing the expression of various antioxidant proteins and oxidases (Madduma Hewage et al., 2021). Under physiological conditions, Nrf-2 is kept in the cytoplasm by binding to its endogenous inhibitor, Keap1. This binding subjects Nrf-2 to Keap1-mediated rapid ubiquitination and subsequent proteasomal degradation (Kubo et al., 2017; Li J. et al., 2019; Gu et al., 2025). Research has elucidated that the Keap1-Nrf-2 pathway effectively protects the liver from lipotoxic damage in MASLD (Sun Z. et al., 2025). Accumulating evidence supports the hepatoprotective role of Nrf-2 activation. For example, a study by Susara et al. revealed that a HFD led to reduced hepatic glutathione (GSH) levels and decreased nuclear accumulation of Nrf-2 (Madduma Hewage et al., 2021). In addition, Nrf-2 knockout mice exhibited more severe hepatic inflammation and fibrosis following CCl_4_ exposure (Lee et al., 2020).

Currently, Rezdiffra is an FDA-approved drug for the treatment of moderate to severe MASH. Rezdiffra reduces hepatic triglyceride levels by modulating genes involved in MB/autophagy, fatty acid β-oxidation, and lipophagy, thereby decreasing lipotoxicity and improving mitochondrial function (Lian et al., 2024). In parallel, numerous natural compounds have shown promising hepatoprotective potential. For instance, the natural compound dioscin functions as a SIRT1 agonist. By activating the SIRT1/PGC-1α pathway, dioscin subsequently upregulates key downstream targets such as Nrf-1, COX−IV, and PPARα. This activation helps to improve mitochondrial function, boost FAO, and reduce inflammation (Meng et al., 2024). Similarly, Cyanidin-3-glucoside (Cy3g) also demonstrates hepatoprotective potential. Its beneficial effects appear to be mediated through improved mitochondrial function in hepatocytes and the upregulation of MB−related genes (including PGC−1α, Nrf−1, and TFAM) (Mogalli et al., 2018). MB agonists have attracted increasing attention as a potential strategy for MASLD. MB agonists reshape hepatocyte energy metabolism networks by targeting key transcription factors including PGC-1α, Nrf-2, and TFAM. The mechanisms of MQC are being increasingly clarified, and multi-target synergistic regulatory strategies are under development. Against this backdrop, interventions centered on MB are emerging as a potential core platform for the next generation of metabolic liver disease therapies.

#### 3.1.2 Regulate mitochondrial dynamics balance

MD equilibrium is a dynamic regulatory state governed by the balance of mitochondrial fusion and fission. This state fundamentally underpins cellular health by sustaining mitochondrial network integrity, supporting efficient energy metabolism, and preserving overall homeostasis (Liu et al., 2024). A hallmark of MASLD pathology is an imbalance in MD, characterized by excessive fission and suppressed fusion. This imbalance drives mitochondrial fragmentation and consequent dysfunction, a state marked by reduced ATP production (Zhang Z. et al., 2025). Consequently, a therapeutic strategy aimed at restoring mitochondrial morphology and function is being explored for MASLD. This approach involves modulating the expression of key fusion (Mfn-1/2, Opa1) and fission (Drp1, Fis1) proteins. The AMPK signaling pathway plays a central role in regulating mitochondrial fusion. Phosphorylated AMPK promotes the expression of fusion proteins including Mfn1, Mfn2, and Opa1 by activating the downstream PGC-1α signaling pathway. This intervention also promotes mitochondrial health by maintaining ultrastructure and homeostasis, upregulating ATP synthesis, and enhancing overall function. Concurrently, it inhibits apoptotic pathways. These combined actions yield significant cytoprotective effects (Gao et al., 2019; Wang M. et al., 2020; Tang et al., 2023). Opa1 is an essential protein for IMM fusion. Beyond this primary role, it is also involved in maintaining cristae structure and protecting cells from apoptotic stimuli. Multiple compounds have demonstrated potential in promoting mitochondrial fusion. For example, the mitochondria-targeted peptide SBT-20 (also known as SS-20) maintains normal mitochondrial structure by reducing fission protein Drp1 expression and increasing fusion protein Mfn2 expression, while simultaneously decreasing ROS generation and maintaining normal ETC function and ATP production (Wang et al., 2022). Another example, MitoQ, as a mitochondria-targeted antioxidant, upregulates fusion protein (Mfn1, Mfn2, Opa1) expression and downregulates fission protein (Drp1, Fis1) expression, maintaining mitochondrial network integrity (Gan et al., 2018).

Research has revealed that excessive mitochondrial fission is an important mechanism in MASLD pathogenesis. Genetic inhibition of mitochondrial fission can alleviate HFD-induced hepatic steatosis. Drp1 acts as a key regulator of mitochondrial fission, and its inhibitor Mdivi-1 can effectively inhibit excessive mitochondrial fission, increase the expression and nuclear translocation of PGC-1α and its downstream transcription factors (Nrf-1/2, TFAM), and promote MB (Ma et al., 2025). Notably, in methionine-choline-deficient diet-induced MASH models, Opa1 knockout prevents megamitochondria formation and liver damage, suggesting the importance of moderate fission regulation for hepatic protection (Yamada et al., 2022).

Studies have shown that prolonged exposure to high-fat, high-carbohydrate diets leads to glucose and lipid metabolic disorders, which can simultaneously downregulate Mfn1 and upregulate mitochondrial Fis1, inhibiting mitochondrial fusion and promoting mitochondrial fission. This rise in mitochondrial fragmentation worsens mitochondrial dysfunction. This compromised function, in turn, drives the development of MASLD (Cho et al., 2022). A growing body of research has elucidated the pivotal role of natural products in regulating MD, particularly in metabolic stress-related hepatic disorders. For instance, alpha-lipoic acid (LA) modulates MD by upregulating fusion genes (Mfn1, Mfn2) and downregulating the fission gene Drp1. This shift in gene expression helps restore cellular bioenergetics following palmitic and oleic acid treatment (Luo and Shen, 2020). *In vitro* steatosis models demonstrate that LA reduces lipotoxicity and directly improves mitochondrial function. In HFD-induced mouse models, puerarin upregulates the expression of mitochondrial fusion-related proteins (Mfn1, Mfn2, Opa1) and downregulates the levels of fission-related proteins (Drp1, Fis1). This promotes mitochondrial fusion, inhibits excessive mitochondrial fission, maintains mitochondrial morphological and structural integrity, and stabilizes the homeostasis of the MQC system. Meanwhile, puerarin remodels the composition of gut microbiota and alters the profile of microbiota-derived short-chain fatty acids. Through the distant regulatory effect of the gut–liver axis, it ameliorates hepatic lipid metabolic disorder and oxidative stress injury, thereby delaying the pathological progression of MASLD (Sun C. et al., 2025). Irisin exerts multiple beneficial effects in MASLD models. It activates the PKA/SIRT3/mTOR pathway, leading to increased expression of fusion proteins (Mfn2, Opa1) and decreased expression of the fission protein Drp1. At the metabolic level, this is accompanied by promoted mitochondrial fatty acid uptake and β-oxidation, along with inhibited lipogenesis. Consistently, in both HFD-induced mouse models and PA-treated HepG2 cells, irisin treatment significantly reduces lipid accumulation (Li et al., 2025). MD imbalance is a core component of MASLD pathogenesis, and precise regulation targeting the Mfn/Opa1 fusion axis and Drp1/Fis1 fission axis provides novel therapeutic perspectives for metabolic liver disease. Current research has evolved from single-target intervention to multi-pathway synergistic regulation, indicating that combined therapeutic strategies based on MD equilibrium will become an important direction for future MASLD precision medicine.

#### 3.1.3 Enhance mitophagy

When mitophagy mechanisms are impaired, hepatocyte energy metabolism becomes compromised. Elimination of damaged mitochondria is important for keeping hepatocyte homeostasis (Zhang C. et al., 2025). Enhancing mitophagy has emerged as one of the core strategies for improving hepatocyte function and alleviating MASLD. Multiple studies have demonstrated that various drugs and natural compounds can effectively activate mitophagy by targeting different signaling pathways, thereby reducing hepatic lipid accumulation, oxidative stress, and inflammatory responses. Notable advancements have been made in multiple studies on regulating the core mechanism of autophagy. Hsu et al. discovered that apigenin, a flavonoid, alleviates hepatic lipid deposition by stimulating the autophagy-mitochondria pathway (Hsu et al., 2021). Mechanistically, apigenin treatment increases the expression of autophagy-related proteins Beclin1, autophagy-related gene 5 (ATG5), ATG7, and LC3II, and promotes autolysosome formation, thereby accelerating fatty acid consumption and reducing intracellular lipid levels (Hsu et al., 2021). Apigenin contributes to hepatic protection by promoting mitochondrial health. It does so through upregulated expression of mitochondrial fusion proteins, which improves network structure and function, thereby aiding in the prevention of lipid accumulation (Hsu et al., 2021). Cordycepin, as reported by Tian et al., counters MASLD by clearing damaged mitochondria via enhanced mitophagy and MB. A key action is the activation of Parkin-mediated mitophagy. Through this pathway, cordycepin works to restore mitochondrial homeostasis and lower oxidative stress, which collectively alleviate disease progression (Tian et al., 2025). Magnoflorine provides protection in MASLD mice, as shown in HFD-induced MASLD mice. Its beneficial effect is mediated by activating mitochondrial autophagy, which concurrently dampens NLRP3 inflammasome activity and clears dysfunctional mitochondria. This dual action results in reduced hepatic steatosis, inflammation, and liver injury (Wang L. et al., 2025). Research in MASLD mouse models indicates that under stress conditions like hypoxia, BNIP3-mediated mitophagy can reduce hepatocyte lipid accumulation, in part by promoting fatty acid β-oxidation. This finding highlights BNIP3 as a potential therapeutic target for intervention (Joaquim and Escobar-Henriques, 2020; Delgado et al., 2024; Tian et al., 2024). Guan et al. discovered that lactoferrin promotes mitophagy in hepatocytes. This enhanced mitophagy facilitates the rapid clearance of mitochondria impaired by lipopolysaccharide. The removal of these dysfunctional organelles thereby reduces the associated surge in ROS levels, leading to decreased oxidative stress and necrosis (Guan et al., 2025). Studies indicate that diosgenin (DIO) can regulate mitochondrial autophagy (mitophagy) in hepatocytes. This regulation is mediated through stimulation of the ERα/AMPK signaling pathway. Enhanced mitophagy via this pathway reduces mitochondrial damage and apoptosis, contributing to hepatoprotection (Xing et al., 2024).

In conclusion, enhancing mitophagy represents a highly promising direction for MASLD treatment. The aforementioned studies indicate that multiple drugs can improve mitochondrial function. Concurrently, these medications are capable of eliminating damaged mitochondria and alleviating hepatic steatosis and inflammation. Enhanced mitophagy, a therapeutic goal in MASLD, can be achieved by either directly targeting core autophagy machinery (LC3, Beclin1) or by activating upstream pathways (PINK1-Parkin, AMPK, and BNIP3). Future research should focus on elucidating the interactions between these pathways and translating these preclinical findings into effective clinical treatment strategies.

### 3.2 Strategies for treating MASLD by indirectly regulating mitochondrial function

#### 3.2.1 Antioxidant strategy

In MASLD, oxidative stress and mitochondrial dysfunction reinforce each other in a vicious cycle. The cycle often begins with oxidative stress damaging mitochondrial function. This impairment specifically disrupts fatty acid β-oxidation. Consequently, FAs accumulate in the liver, driving the development of hepatocyte steatosis (Chen et al., 2025). The steatotic hepatocyte-mitochondria dysfunction cycle further intensifies oxidative stress, a state defined by ROS production exceeding clearance capacity. Excessive generation of ROS initiates a cascade of damage. This begins with the oxidation of critical biomacromolecules (DNA, lipids, and proteins). At the cellular level, such oxidative damage undermines structural integrity and disrupts normal function. Collectively, these impairments progress to overt liver injury (Chiş et al., 2023). Consequently, enhancing antioxidant defense has emerged as a potential strategy for MASLD treatment. At physiological concentrations, ROS act as important signaling molecules. They do so by participating in the regulation of essential processes, including cell cycle control and intercellular communication. However, once produced in excess, ROS exhibit significant cytotoxicity, triggering oxidative stress and causing cellular damage, DNA mutations, and even cell death (Touyz et al., 2020). Through its effects on lipids, proteins, and DNA, oxidative stress participates during the pathogenesis and progression of various degenerative disorders, including MASLD, diabetes, cardiovascular diseases, and neurodegenerative diseases (Park et al., 2022). Studies have shown that in certain circumstances, the use of exogenous antioxidants and ROS scavengers can partially alleviate the pro-apoptotic effects induced by lipid-induced toxicity. Given this close relationship between antioxidant intervention and FA-induced pro-apoptotic effects, endogenous antioxidant pathways have become a research focus. For instance, stimulation of the Nrf-2 pathway has been confirmed to significantly reduce oxidative stress and inflammation levels in MASLD models (Clare et al., 2022; Su et al., 2023; Wang L. et al., 2024). Furthermore, when cells are exposed to oxidative stress, mitochondria initiate a series of defense mechanisms, such as recruiting antioxidants, activating DNA repair systems, and promoting the folding and degradation of misfolded proteins, to maintain normal mitochondrial function (Ramanathan et al., 2023).

During recent years, extensive research has centered on the antioxidant effects of traditional Chinese herbs and their active components, confirming their ability to effectively inhibit ROS production and accumulation while improving hepatocellular lipid deposition (Xiong et al., 2017; Xu M. et al., 2019; Xu et al., 2020; Zhou X. et al., 2024; Zhou et al., 2025) (**Table 1**). For example, Polygonum cuspidatum extract mitigates the progression of HFD-induced MASLD by maintaining mitochondrial ultrastructural integrity, upregulating SOD and GSH activities, reducing MDA levels, scavenging ROS, and inhibiting oxidative stress, thereby correcting lipid metabolism disorders (Yu et al., 2022). Carotenoids remodel the hepatic immune microenvironment by modulating liver macrophage polarization (inhibiting M1 and promoting M2 phenotypes), thereby reducing the release of pro-inflammatory cytokines such as TNF-α, IL-1β, and IL-6. Concurrently, they maintain hepatic metabolic and immune homeostasis through their potent antioxidant and anti-inflammatory activities, ultimately halting the progression of MASLD to MASH and fibrosis (Ni et al., 2016). Furthermore, Ramulus Mori (Sangzhi) alkaloids (SZ-A), an active ingredient extracted from mulberry twigs, ameliorate lipid metabolism disorders, enhance systemic antioxidant capacity, and inhibit hepatic collagen deposition and fibrosis in HFD-induced MASLD mice by regulating the PGC-1α/PPARα and Keap1/Nrf2 signaling axes (Chen et al., 2022; Zhang et al., 2024). In addition to alkaloids with antioxidant properties, flavonoid compounds represent another major class of natural products that show significant potential in antioxidant defense (Xu et al., 2022). Liquiritin and isoliquiritin, the key flavonoid antioxidants in licorice, exert a core protective effect against oxidative stress and lipid peroxidation in cells (Zhang E. et al., 2020; Shang et al., 2023; Wang J. et al., 2024). Studies in animal models have confirmed several beneficial actions of licorice. These include the reduction of hepatic steatosis, modulation of lipid metabolism, and overall improvement in liver function (Cheng et al., 2019). Quercetagetin (QG), another key flavonoid derivative, is both widely abundant in botanical sources and possesses potent antioxidant potential. This dual characteristic enables QG to effectively neutralize surplus ROS and alleviate oxidative cellular damage (Wu et al., 2023). QG activates the Keap1-mediated Nrf-2/ARE signaling pathway. This activation leads to two direct biochemical effects: enhanced antioxidant enzyme activity and reduced malondialdehyde (MDA) levels. As a result, the body’s overall antioxidant status is strengthened, which contributes to improved intestinal morphology and nutrient absorption (Wu et al., 2022). Furthermore, QG possesses diverse biological properties encompassing antiviral, antidiabetic, and antihyperlipidemic effects (Kızıldağ et al., 2024). To summarize, antioxidants exert effective intervention on hepatic steatosis via diverse mechanisms. Mainly including suppressing *de novo* lipogenesis in hepatocytes, enhancing the clearance of oxidative stress products, and preserving mitochondrial functional integrity. Therefore, these antioxidants exhibit significant potential for application in the prophylaxis and management of MASLD.

**Table 1:** The application of antioxidants in liver diseases.

Antioxidant	Mechanism	Models
Berberine (Liu et al., 2025)	Berberine mitigates HFD-induced NAFLD in mice by activating SIRT3 and promoting mitochondrial β-oxidation.	NAFLD
Silybin (Xu X. et al., 2019)	Silybin mitigates MASH-induced liver fibrosis by restraining succinate production and its extracellular release.	MASH
Silibinin (Yang X. et al., 2025)	Silibinin maintains mitochondrial homeostasis by upregulating Opa1 expression in hepatocytes. oxidative stress ↓, lipid accumulation ↓	HepG2 cells/MASLD
Novel silybin derivative A2 (Sui et al., 2025)	Novel silybin derivative A2 showed excellent activity in restraining lipid accumulation, antioxidation, anti-inflammation, and antifibrosis.	MASH
Pueraria lobata antioxidant extract (Dong et al., 2024)	Pueraria lobata antioxidant extract can reduce TC and TG. Specifically, it alleviates cellular oxidative stress by restraining ROS release, increasing MMP, and inhibiting apoptosis. TC ↓, TG ↓, ROS ↓, MMP↑	NAFLD
Curcumin (Lee DE. et al., 2019)	Curcumin treatment reduces the severity of hepatic steatosis. At the molecular level, it acts by attenuating the activation of the pro-inflammatory NF-κB signaling pathway, specifically by reducing its dependence on O-GlcNAcylation.	NASH mic
Acerola polysaccharides (Hu et al., 2020)	Acerola polysaccharides counteract HFD-induced NAFLD via the regulation of lipogenesis, reduction of inflammation and oxidative stress, and enhancement of mitochondrial function. mitochondrial ATP content ↑, mitochondrial complex I, IV, and V activity ↑, mitochondrial beta-oxidation ↑	HFD-induced NAFLD
Polygonatum kingianum (Yang et al., 2019)	Polygonatum kingianum alleviated HFD-induced NAFLD by promoting mitochondrial functions. MDA content ↓, activities of GSH-PX, SOD, Na+-K+-ATPase, and complex I and II ↑	HFD-induced NAFLD
Carnosol (Geng et al., 2021)	Carnosol exerts protective effects against hepatic injury, steatosis, and apoptosis in NAFLD. These benefits are mediated through multiple cellular actions: it helps balance mitochondrial fusion and fission, maintains the MMP, and reduces oxidative stress. MPP ↑, ATP level ↑, mitochondrial fusion ↑, mitochondrial fission ↓, H2O2 level ↓, mtROS level ↓	AML-12 cells, HFD-induced NAFLD
Diosgenin (Zhong et al., 2022)	Diosgenin ameliorated type II diabetes-associated nonalcoholic fatty liver disease through inhibiting *de novo* lipogenesis and improving FA oxidation and mitochondrial function in rats. mitochondrial fusion ↑, mitochondrial fission ↓, ROS level ↓	Type II diabetes-associated nonalcoholic fatty liver disease (D-NAFLD)
Baicalin (Gao et al., 2022)	In the 3D NAFLD model, baicalin reduces oxidative stress through its intrinsic antioxidant activity. This reduction in stress helps protect mitochondrial integrity, which in turn contributes to the inhibition of apoptosis. ROS level ↓, MMP ↑	NAFLD

#### 3.2.2 Regulate bile acid metabolism

Bile acids (BAs) are an important class of endogenous cholesterol-derived metabolites in the liver through two major pathways, playing crucial roles in bile secretion and intestinal uptake of dietary lipids and fat-soluble vitamins (Schneider et al., 2018). Cholesterol is primarily catalyzed through the classical BA synthesis pathway to generate cholic acid (CA) and chenodeoxycholic acid (CDCA). Cytochrome P450 family 7 subfamily A member 1 (CYP7A1) and Cytochrome P450 family 8 subfamily B member 1 (CYP8B1) are key enzymes in the classical BA synthesis pathway (Li and Lu, 2018). In the alternative pathway, cholesterol is converted to 27-hydroxycholesterol (27HC) via sterol 27-hydroxylase (CYP27A1), which is ultimately metabolized to CDCA (Miyazaki et al., 2020). CYP27A1 and cytochrome P450 7B1 (CYP7B1) are responsible for cholesterol hydroxylation and oxidation in the alternative pathway. Studies have found that under pathological conditions such as cirrhosis, the release of pro-inflammatory cytokines can inhibit CYP7A1 transcription, thereby suppressing the classical pathway. Consequently, the alternative pathway becomes the predominant route for BA synthesis (Li and Lu, 2018). Notably, in HFD-induced MASLD mice, inhibition of hepatic cholesterol-25-hydroxylase (Ch25h) attenuates CYP27A1-dependent BA biosynthesis and secretion, thereby exacerbating hepatic steatosis (Dong et al., 2022).

BA metabolic dysregulation and the resulting cytotoxic effects are strongly correlated with impaired mitochondrial function. Several investigations have demonstrated that increased BA concentrations exert toxic effects on cells, as they can modulate mitochondrial function to varying degrees. In isolated liver mitochondria, BAs such as deoxycholic acid (DCA) induce dose-dependent membrane depolarization and opening of the mPTP (Dong et al., 2022). Research has revealed that glycochenodeoxycholic acid (GCDCA) not only downregulates PGC-1α gene expression and impairs MB, but also inhibits Cyt C activity, further aggravating mitochondrial dysfunction (Alqabandi et al., 2025). Furthermore, multiple therapeutic strategies have emerged targeting disorders of bile acid metabolism and their associated mitochondrial toxicity. Wang et al. found that sorghum bran extract (SBE) modulates both hepatic bile acid synthesis and mitochondrial function. It does so respectively by elevating expression of BA synthetic enzymes and by activating PPARδ to upregulate FAO/OXPHOS genes (Wang Y. et al., 2025). Luo et al. found that the traditional Chinese medicine formula Yinzhihuang (YZH) alleviates alpha-naphthyl isothiocyanate (ANIT)-induced liver injury and fibrosis. YZH not only reduces bacterial translocation from the gut to liver tissue by preserving intestinal barrier integrity, but also promotes BA excretion by enriching BA metabolism-related probiotics (Luo et al., 2025). Ursodeoxycholic acid (UDCA), as a hepatoprotective agent, prevents BA attack on the liver by inhibiting the binding of lipids with hydrophobic BAs on the hepatocyte granular membrane, reduces mitochondrial cytochrome release, decreases mitochondrial membrane permeability, thereby protecting hepatocytes and preventing apoptosis (Beuers and Trampert, 2022). Interestingly, in hepatocyte-specific OPA1-deficient mice, impaired hepatic mitochondrial fusion, while reducing physical interactions between liver mitochondria-peroxisomes-ER and weakening dietary lipid absorption and BA secretion, paradoxically protected mice from HFD-induced metabolic dysfunction through this metabolic reprogramming (Da Dalt et al., 2024). Finally, in cholestatic liver, impaired bile formation or excretion leads to BA accumulation in systemic blood and hepatocytes. When BA concentrations exceed the binding capacity of cytoplasmic binding proteins in hepatocytes, BAs directly damage mitochondria, inducing hepatocyte apoptosis or even necrosis, indicating that maintaining BA homeostasis is crucial for hepatocyte survival (Palmeira and Rolo, 2004).

#### 3.2.3 Regulate gut microbiota

The liver is the organ most functionally connected to the intestine, with approximately 75% of hepatic blood flow is supplied by the portal vein, which collects blood from the gut (Wan et al., 2019; Bellanti et al., 2023; Ding et al., 2024). Portal venous blood contains not merely metabolites but also gut-absorbed bacterial metabolic derivatives, as well as bacterial components or even viable bacteria that may appear during intestinal barrier dysfunction. This mixture directly reaches the liver, where the gut microbiota and its metabolites have emerged as key regulators of mitochondrial metabolism, MB, and redox balance (Huang et al., 2022). Research indicates that the gut microbiota in MASLD patients can produce significant quantities of ethanol. Transported via the portal circulation directly to the liver (bypassing first-pass effect), this endogenous ethanol accumulates within the liver, where it acts as a direct hepatotoxin, contributing to the development of steatosis and inflammation (Meijnikman et al., 2022).

The gut microbiota is a complex consortium of microorganisms, encompassing bacteria, viruses, and fungi. This microbial community is integral to maintaining intestinal homeostasis, and its dysregulation is implicated in various diseases (Jackson and Theiss, 2020). The gut microbiota has been shown to regulate key factors in MB, including transcriptional coactivators, transcription factors, and enzymes. The gut microbiota and its metabolites, including short-chain fatty acids and secondary BAs, help alleviate intestinal inflammation. They do this by attenuating TNF-α-mediated immune responses and inhibiting the activation of inflammasomes like NLRP3. By reducing inflammation in this manner, they indirectly support the maintenance of host energy metabolism and ROS homeostasis (Clark and Mach, 2017). Evidence for interactions between the gut microbiota and hepatic mitochondria also derives from studies treating MASLD with novel functional foods or fecal microbiota transplantation, which target the microbiota and exert effects on mitochondrial function (Zhang et al., 2022). Zhou et al. demonstrated that in HFD-induced NASH mouse models, fecal microbiota transplantation could alleviate steatohepatitis by regulating the gut microbiota composition (Zhou et al., 2017). Sodium butyrate, a gut-derived short-chain fatty acid, preserves mitochondrial functional homeostasis *in vivo*, while attenuating hepatic inflammatory responses and oxidative stress (Xia et al., 2025). Xylo-oligosaccharides have been explored for the treatment of HFD-induced MASLD. Xylo-oligosaccharides increases the abundance of Faecalibacterium prausnitzii, alleviates intestinal inflammation, elevates hepatic β-HAD activity and mitochondrial complex I level, enhances OXPHOS, improves mitochondrial respiratory capacity, and promotes ATP production (Gulhane et al., 2016). In addition, Xylo-oligosaccharides reduces the levels of tyrosine and isovalerate in MASH conditions, thereby facilitating hepatic lipid oxidation (Gitto et al., 2018). In conclusion, targeted regulation of gut microbiota composition to improve mitochondrial metabolic function has evolved as a promising novel strategy for MASLD treatment.

#### 3.2.4 Nanoparticle-targeted therapy strategies

The pathogenesis of MASLD has been extensively investigated, and mitochondrial functional disturbance has been established as a core driver of disease progression. Nanotechnology, with its precise targeting and controlled release capabilities, offers a promising platform for developing interventions to restore mitochondrial function and treat MASLD. Currently, various mitochondria-targeting nanoparticles have been designed to effectively alleviate hepatocellular lipid deposition and oxidative injury by scavenging ROS, improving energy metabolism, and modulating autophagy (**Table 2**). Gao’s research team designed a mitochondria-targeted black phosphorus/cerium nanozyme (TBP@CeO2) with dual functions of antioxidation and energy regulation. *In vivo* studies demonstrated that TBP@CeO2 nanozyme can enhance hepatic complex II activity and mitochondrial function, effectively reduce ROS accumulation and maintain adequate ATP production, thereby helping to protect liver tissue from oxidative damage (Gao et al., 2025). Additionally, Zhang et al. engineered a microenvironment-responsive programmable nanotherapeutic agent (CsA@Dex-Gal/TPP). This nanoformulation improves mitochondrial dysfunction in steatotic HepG2 cells by inhibiting mPTP opening, maintaining MMP, and diminishing ROS generation. CsA@Dex-Gal/TPP nanoparticles amassed in the liver significantly ameliorate hepatic lipid deposition in MASLD mice by restoring mitophagy and correcting glucose and lipid metabolic abnormalities (Zhang J. et al., 2025). Cai et al. discovered that orally administered inorganic nanoparticles target and accumulate within the hepatocellular ER-mitochondria system. By inducing mitochondria-derived low levels of ROS to activate the Nrf2-Ces2h signaling axis, these nanoparticles exert dual effects: on one hand, they promote hepatic lipid ester hydrolysis and enhance mitochondrial FAO to reduce lipid accumulation; on the other hand, they mitigate lipotoxicity and hepatic steatosis by clearing ROS via Ces2h and maintaining mitochondrial redox homeostasis (Cai et al., 2023). The aforementioned studies demonstrate the diversified strategies of nanoparticles in targeting mitochondria for MASLD treatment. Nanotherapy not only effectively alleviates hepatocellular metabolic abnormalities and lipid accumulation but also provides potential translational directions for treating mitochondria-related liver diseases. Future research needs to further optimize targeting efficiency and biosafety to advance the clinical application of nanoformulations.

**Table 2:** Nanoparticle diagnosis and treatment of liver diseases.

Name of nanoparticles	Regulatory mechanisms/pathways	Target site	Cell lines/Disease types
Clam-derived exosome-like nanovesicles (Hu et al., 2024)	Amelioration of AIL damage by protecting mitochondrial dysfunction and suppressing the mitochondrial apoptotic pathway. ATP levels ↑, mitochondrial complex I activity ↑, mitochondrial ROS production ↓.	Mitochondrial	HepG2 cells/Alcoholic liver disease (ALD)
Mitochondria-targeted esculetin (Singuru et al., 2023)	Mito-Esc reverses palmitic acid-induced mitochondrial superoxide production, mitochondrial membrane depolarisation and lipid accumulation in HepG2 cells by activating the AMPK-SIRT1 pathway.	Mitochondrial	NAFLD/NASH/HepG2 cells
high-PC-content liposome-loaded curcumin (Wang et al., 2026)	Mechanistically, hPLipo/Cur reduces Nrf-2 degradation, promotes the nuclear translocation of Nrf-2 and the expression of downstream antioxidant genes. The activated Nrf-2 pathway reduces cellular oxidative stress and mitochondrial ROS production, thereby reducing the accumulation of lipid peroxides and inhibiting the progression of steatohepatitis. ROS levels ↓,	Liver	NASH
ER-ZS (Zeng et al., 2024)	ER-ZS achieves precise ER targeting via ER-targeted functional groups and exhibits specific fluorescence response to viscosity, enabling targeted diagnosis of NAFLD through liver imaging based on elevated hepatic ER stress.	ER	NAFLD
EC@PLGA-PEG-GAL NPs (Han et al., 2025)	EC@PLGA-PEG-GAL NPs effectively restored mitochondrial structure and mitochondrial membrane potential, whilst reducing the production of mitochondrial ROS. EC@PLGA-PEG-GAL NPs upregulated the expression of genes associated with mitochondrial biogenesis and favourably modulated the expression of genes related to mitochondrial dynamics, thereby comprehensively improving mitochondrial function, maintaining mitochondrial homeostasis, and slowing the progression of MASLD. ROS levels ↓, MMP ↑	liver tissues	MASLD mice model
Astaxanthin@ triphenylphosphonium- whey protein isolate- galactose (Che et al., 2023)	Alleviate blood lipid disorders, maintain normal hepatic physiology, and notably attenuate hepatic lipid accumulation via lipid metabolism modulation.	Hepatic parenchymal cell and mitochondrial	HepG2 cells/NAFLD mice model
Blueberry-derived exosomes-like nanoparticles (Zhao et al., 2022)	Reducing lipid droplet aggregation and oxidative stress, ameliorating insulin resistance, and improving hepatocellular dysfunction. ROS levels ↓, MMP ↑	Mitochondrial	HepG2 cells/NAFLD
Pomegranate-derived exosome-like nanovesicles (Hou et al., 2023)	Oral dosing of Pomegranate-derived exosome-like nanovesicles accumulated in the liver of mice and effectively alleviated HFD-induced NAFLD by improving mitochondrial function. ATP levels ↑, mitochondrial complex I activity ↑, ROS levels ↓, MDA levels ↓	Mitochondrial	HepG2 cells/NAFLD
Triptolide liposome (Zhou L. et al., 2024)	Inhibit the growth of Hepatocellular Carcinoma. ROS levels ↑, MMP ↓	Mitochondrial	Huh-7 cells/Hepatocellular Carcinoma
Lactobionic acid - 2-hydroxypropyl-β-cyclodextrin - Astaxanthin (Hua et al., 2023)	The Lactobionic acid - 2-hydroxypropyl-β-cyclodextrin - Astaxanthin could favorably prevent depolarization of the mitochondrial membrane and early cell apoptosis. ROS levels ↓, MMP ↑.	Liver	HepaRG cells
Astaxanthin@sea cucumber peptide-whey protein isolate-galactooligosaccharides-triphenylphosphonium (Lv et al., 2025)	antioxidant capacity ↑, lipid accumulation ↓, ROS levels ↓, MMP ↑	Hepatocytes and mitochondria	Human normal liver cells (L-O2)/Alcoholic liver injury (ALI)

#### 3.2.5 Lifestyle intervention: diet and exercise

Lifestyle intervention represents the current first-line strategy for preventing and managing MASLD and its progression to MASH, primarily encompassing low-calorie diets, increased physical activity, and weight reduction (Petroni et al., 2021). In the absence of approved specific pharmacological agents, the combination of dietary control and exercise has been demonstrated to effectively reduce intrahepatic fat content, ameliorate, and even reverse hepatic steatosis (Kwon et al., 2024). Recent studies have further revealed that such interventions not only exert effects through weight loss but also directly modulate mitochondrial function, ER stress, and antioxidant pathways, thereby improving hepatic metabolic status through multiple mechanisms.

Exercise is recognized as a core non-pharmacological strategy for managing MASLD. In the progression of MASLD, physical activity and exercise can exert antioxidant and anti-inflammatory effects by modulating mitochondrial function, thereby reducing fatty accumulation in the liver and lowering body weight. Specifically, exercise plays a hepatoprotective role by diminishing intrahepatic fat deposition, inhibiting inflammatory responses and oxidative stress, as well as improving ER homeostasis, insulin sensitivity and mitochondrial function (Romero-Gómez et al., 2017). In the MASLD zebrafish model, regular exercise synchronously modulates mitochondrial biogenesis, mitochondrial fusion and fission, energy metabolism, and mitophagy via activating the AMPK/SIRT1/PGC-1α signaling pathway, thereby restoring the homeostasis of MQC. This further alleviates lipid deposition, oxidative stress, inflammation and fibrosis in liver tissues of zebrafish, and effectively blocks the occurrence and progression of MASLD (Zou et al., 2023). In the leptin-deficient obese mouse model, moderate-intensity aerobic exercise upregulates PGC-1α to promote mitochondrial biogenesis and enhance the activities of key enzymes involved in hepatic oxidative metabolism. It also repairs mitochondrial dysfunction and ameliorates redox imbalance, thereby relieving the pathological progression of MASLD in obese mice (Fernandes et al., 2020). The above two animal models confirm that regular exercise can achieve hepatoprotection and delay the progression of MASLD by regulating mitochondria-related signaling pathways.

Studies have demonstrated that low-carbohydrate diets are superior to traditional low-fat diets in reducing hepatic fat content (Haufe et al., 2011). As a typical healthy dietary pattern, the Mediterranean diet is characterized by a low-carbohydrate composition along with increased intake of monounsaturated fatty acids and ω-3 fatty acids, which can effectively reduce hepatic fat deposition in patients with MASLD (Trovato et al., 2015). Caloric restriction preserves mitochondrial function and prevents excessive mitophagy by inhibiting fission and promoting fusion to maintain an elongated network (Aon et al., 2016). In addition, low-calorie and low-carbohydrate diets markedly alleviate hepatic lipid accumulation and decrease hepatic fat fraction in individuals with MASLD (Haley et al., 2025). Combined intervention of dietary modification and regular exercise exerts a synergistic therapeutic effect. Dietary intervention paired with exercise may further optimize the management effectiveness of MASLD, particularly in obese-related phenotypes (Vidal-Cevallos et al., 2024). Combined interventions exert multifaceted effects: they improve clinical parameters (weight, hepatic fat) and enhance molecular functions (FGF21 secretion, UPRmt, mitochondrial homeostasis), ultimately delaying MASLD progression (Yuan et al., 2025). A deep understanding of the mechanisms behind combined lifestyle interventions will be crucial for developing tailored, effective non-drug treatments for MASLD.

#### 3.2.6 Others

For the disorder of the MQC system, various corresponding therapeutic drugs have been developed. Additionally, studies have revealed that treatments such as cardiolipin stabilizers, NAD^+^ enhancers, AMPK activators, and mitochondrial autophagy enhancers have provided new potential targets for disease treatment (**Table 3**). Cardiolipin is a mitochondrial-specific phospholipid that is crucial for the functional integrity of the mitochondrial inner membrane. Cardiolipin can shape the structure of the mitochondrial inner membrane, stabilize the morphology of cristae, and ensure the normal operation of the respiratory chain complex (Konovalova et al., 2023). At the same time, it can maintain the membrane potential and stability of the mitochondria, and regulate the fluidity of the membrane (Fukumoto et al., 2019). The formation and maintenance of cristae in the mitochondria of eukaryotic cells require the collaborative action of OPA1, F1-F0 ATP synthase and MICOS complex. Cardiolipin can stabilize F1-F0 ATP synthase and MICOS complex, thereby promoting the formation and stability of cristae (Acehan et al., 2011; Kondadi et al., 2020). Based on this, cardiolipin stabilizers can target and regulate the function of cardiolipin, improve mitochondrial structure and functional abnormalities, and have potential therapeutic value. NAD^+^ enhancers exert therapeutic effects by regulating the NAD^+^ metabolic pathway. NAD+ is synthesized by nicotinamide mononucleotide adenylyltransferase using nicotinamide mononucleotide and ATP as substrates (Magni et al., 2004). Supplementation of NAD^+^ can enhance the activity of SIRT1. This process can promote MBs and inhibit the inflammatory signal transduction mediated by NF-κB2 (Imai and Guarente, 2014). AMPK activators can regulate MD and autophagy processes by activating the AMPK signaling pathway. Under the influence of stress factors such as exercise, AMPK enhances autophagy by promoting mitochondrial division, thereby eliminating dysfunctional mitochondria and maintaining the homeostasis of the mitochondrial population (Zhang and Lin, 2016). Mitochondrial autophagy enhancers mainly function by activating the mitochondrial autophagy pathway. Among them, the mitochondrial autophagy pathway mediated by PINK1-Parkin is the key regulatory pathway. Studies have shown that activating the mitochondrial autophagy mediated by PINK1-Parkin can improve fat deposition and mitochondrial damage in liver cells of MASLD patients (Liu et al., 2018; Liao et al., 2025). Furthermore, cholesterol within mitochondria acts as an auxiliary regulatory factor. It can stabilize the mitochondrial membrane structure, inhibit the opening of the mPTP, and reduce the release of mitochondrial components such as cytochrome c, thereby enhancing the cell’s anti-apoptotic ability and indirectly assisting in the implementation of mitochondrial-related therapeutic strategies (Yue et al., 2023). Apart from the aforementioned pharmacological agents that exert protective effects on mitochondrial function, a wide range of medications have been adopted in clinical settings for the prevention and treatment of MASLD and MASH. These drugs regulate mitochondrial homeostasis by triggering mitochondrial signaling pathways, thereby improving hepatic lipid metabolism, suppressing inflammatory responses and inhibiting liver fibrosis. We have summarized their latest clinical trial progress and documented therapeutic efficacy in **Table 4**. Among them, metformin, vitamin E, liraglutide and semaglutide have been approved. Resveratrol, betaine and pentoxifylline have not yet been approved for liver disease indications and are only involved in multiple NCT national clinical trials.

**Table 3:** Potential therapeutic targets regulating mitochondrial function.

Classification	Types of drugs	Mechanism
Cardiolipin stabilizers	CMP3013	CMP3013, an α-helical amphiphilic peptide containing cyclohexylalanine, α-helical amphiphilic peptide, has high selectivity for cardiolipin, protecting mitochondrial structure and enhancing ATP production (Shin et al., 2024).
	XJB-5-131	XJB-5-131, a mitochondrial-targeted ROS scavenger, inhibits cardiolipin oxidation damage (Liu et al., 2022).
	SS-31	SS-31, stable cardiolipin, maintains the integrity of mitochondrial cristae, preserves the structure of mitochondria, and enables normal NAD^+^ energy metabolism (Birk et al., 2014).
	Cyclosporine A	Cyclosporine A is a potent and widely used inhibitor of mPTP, which can prevent cytochrome c from being released from the mitochondria and reduce the release of mtDNA (Xian et al., 2022).
	Cholesterol	Cholesterol, liposome membrane stabilizer, regulates membrane fluidity, reduces cardiolipin oxidation (Yue et al., 2023).
NAD boosters	Niacin	Niacin, also known as vitamin B3 or nicotinic acid, is a necessary trace nutrient required for the synthesis of NAD. It is the most effective drug for increasing HDL-C, reducing triglyceride levels by 20% to 35% (Nabi et al., 2013).
	Nicotinamide mononucleotide	Nicotinamide mononucleotide is the precursor of NAD+, and supplementing this substance can increase the concentration of NAD+ (Zhao et al., 2026).
	Nicotinamide mononucleotide adenylyl transferase 3	Nicotinamide mononucleotide adenylyl transferase 3 can catalyze the biosynthesis of NAD+ and is located in the mitochondrial matrix (Nikiforov et al., 2011).
	Tryptophan	Tryptophan is the raw material for the *de novo* synthesis pathway and serves to replenish NAD reserves under stress conditions (Koch-Nolte et al., 2011).
AMPK activators	Metformin	Metformin inhibits complex I of the respiratory chain, thereby indirectly increasing the ratio of AMP to ATP, and thereby activating AMPK. The activated AMPK can also reduce the synthesis of fatty acids and cholesterol, and promote the oxidation of fatty acids in the liver (Lim et al., 2010).
	A-769662.	A769662 directly activates the β subunit and inhibits the dephosphorylation process of AMPK, thereby maintaining its activated state. The phosphorylation level at the Thr-172 site of the AMPK protein increases, thereby promoting the mitochondrial biosynthesis and autophagy processes mediated by PGC-1α (Yerra et al., 2017).
	Acadesine	Acadesine (AICAR (5-aminoimidazole-4-carboxamide ribonucleotide), as an AMP analogue, activates the α subunit of AMPK by increasing the intracellular ratio of AMP/ATP. Treatment of iAT2I73T cells with 1 mM AICAR for 24 hours can stimulate mitochondrial biosynthesis and autophagy (Liu et al., 2021b; Rodríguez et al., 2025).
	Mangiferin	Mangiferin increases the AMP/ATP ratio in cells, thereby directly activating the phosphorylation of AMPK at the Thr172 site. The activated AMPK upregulates the expression of CD36 protein, enhancing the ability of liver cells to take up peripheral free fatty acids and reducing the content of free fatty acids in peripheral blood (Niu et al., 2012).
	Resveratrol	Resveratrol inhibits phosphodiesterases, thereby activating AMPK and promoting MB (Bitterman and Chung, 2015).
Mitochondrial autophagy enhancers	Urolithin A	Urolithin A can promote mitochondrial autophagy through the PINK1/TAX1BP1 pathway, and remove damaged mitochondria through autophagy (Fan et al., 2022).
	MTX115325	USP30 is a mitochondrial-specific deubiquitinating enzyme that can inhibit the mitochondrial autophagy process. The USP30 inhibitor MTX115325 can enhance mitochondrial autophagy (Fang et al., 2023).
	Corn peptides	Corn peptide alleviates NAFLD by mediating mitochondrial autophagy through PINK1/Parkin. Moreover, corn peptide can also alleviate the histopathological changes, lipid metabolism disorders and mitochondrial damage induced by HFD (Yao et al., 2023).
	Quercetin	Quercetin activates PINK1-Parkin-mediated mitochondrial autophagy, which can alleviate fat deposition and mitochondrial damage in liver cells (Liu et al., 2018).

**Table 4:** The drugs in clinical trials and their mechanisms of action.

Drugs	National Clinical Trial numbers	Mechanism of action
Vitamin E	NCT00063622	Restoration the hepatic glutathione level. Improved steatosis and inflammation (Sanyal et al., 2010).
Metformin	NCT02696941	Activation of AMPK signaling. Improved parenchymal inflammation and cellular injury (Loomba et al., 2009).
Resveratrol	NCT02030977	Activation of mitochondrial biogenesis and mitochondria-located antioxidant enzymes. Improved hepatic steatosis (Faghihzadeh et al., 2014).
Betaine	NCT03073343	Restoration of hepatic mitochondrial glutathione and S-adenosyl methionine. Improved hepatic steatosis (Abdelmalek et al., 2009).
Pentoxifylline	NCT00267670	Increasing Nrf-2 and PGC-1α through the cAMP–CREB pathway. Improved steatosis, inflammation, and fibrosis (Zein et al., 2011).
Liraglutide	NCT02147925	Enhancing mitochondrial architecture through the SIRT1/SIRT3 signaling. Improved hepatic steatosis (Armstrong et al., 2016).
Exenatide	NCT02303730	Enhancing mitochondrial architecture through the SIRT1/SIRT3 signaling (Liu et al., 2021a).
Semaglutide	NCT02970942	Enhancing mitochondrial architecture through the SIRT1/SIRT3 signaling. Improved hepatic fibrosis and reduced liver-enzyme levels (Newsome et al., 2021).
Randomized	N0192119052 ISRCTN: 10319160	Activate AMPK to promote the β-oxidation of fatty acids in the liver and reduce lipid accumulation (Aithal et al., 2008).
Lanifibranor	NCT03008070	PPAR agonists. Improve insulin resistance and regulate glucose and lipid metabolism. Lanifibranor can improve insulin resistance, regulate glucose and lipid metabolism, and exert anti-inflammatory and anti-fibrotic effects (Francque et al., 2021).

## 4 Conclusion and prospect

Mitochondria serve as the cellular powerhouse, central to energy metabolism and a primary source of oxidative stress. Therefore, the maintenance of mitochondrial functional homeostasis is critically involved in the onset and progression of MASLD. Given this centrality to MASLD pathogenesis, MQC has emerged as a critical focal point. In the course of MASLD, hepatocyte mitochondria experience a significant decline in function. This impairment is evident across key areas of mitochondrial physiology and compromises multiple aspects of the MQC system. Key characteristics of this impairment include abnormal mitochondrial morphology, reduced OXPHOS efficiency, diminished ATP production, decreased MMP, excessive ROS generation, compromised MB, imbalanced fission-fusion dynamics, and decreased mitophagy. These abnormalities not only result in insufficient energy supply to hepatocytes but also exacerbate lipid metabolic disorders, inflammatory responses, and fibrotic processes through oxidative stress-induced damage. Currently, most of the MQC targeted intervention studies focus on AMPK activators, small molecule mitochondrial autophagy enhancers, and natural active components, providing preliminary ideas for the prevention and treatment of MASLD. However, there is still a limitation of a single intervention method.

With the rapid development of emerging experimental technologies, the research on MASLD targeting MQC has ushered in a new breakthrough opportunity. Mitochondrial transplantation therapy can restore liver cell metabolism by transplanting healthy mitochondria, inhibit the activation of hepatic stellate cells, and effectively reduce lipid deposition and liver fibrosis, demonstrating significant clinical translational value; the deep integration of artificial intelligence and mitochondrial biomarkers has opened up a new path for the clinical precise diagnosis of MASLD. At the same time, new intervention methods targeting MQC are constantly expanding. Using PGC-1α, PINK1, Parkin, Mfn2 as the target genes, and employing AAV liver-targeted gene overexpression, CRISPR gene editing technologies, as well as RNA-targeted therapeutic strategies such as siRNA silencing of negative regulatory genes, miRNA mimics/inhibitors, and antisense nucleic acids, can precisely regulate the entire process of MQC at the transcriptional, post-transcriptional, and genetic levels, providing a new non-drug intervention idea for MASLD, and is expected to make up for the singleity of existing intervention methods.

However, the current research on MASLD targeting MQC still faces numerous challenges: the regulatory mechanism of the interaction between MQC and genetic factors has not been fully elucidated, the dynamic balance relationship between cell pyroptosis and mitochondrial autophagy still needs to be further explored, and the clinical translation of MQC targeting strategies is hindered by the unclear mechanism of action and the limitations of single-target intervention. Future research should focus on the above key issues. On one hand, it should deeply analyze the core molecular mechanisms of MQC dysregulation, clarify the interaction between genetic factors, cell pyroptosis and MQC; on the other hand, it should further optimize the application of emerging technologies, systematically explore the potential of new intervention strategies such as gene therapy and RNA-targeted therapy, and promote the development of multi-target and multi-dimensional intervention schemes, breaking the limitations of single-target intervention. Ultimately, the efficient transformation of basic research results into clinical applications should be achieved, providing more targeted theoretical basis and technical support for the precise prevention, diagnosis and treatment of MASLD.

## 5 Nomenclature

MQC, Mitochondrial quality control; MB, Mitochondrial biogenesis; MASLD, Metabolic dysfunction-associated steatotic liver disease; NAFLD, Nonalcoholic fatty liver disease; FAO, Fatty acid oxidation; TG, Triglyceride;; TC, Total cholesterol; TCA cycle, Tricarboxylic acid cycle; OXPHOS, Oxidative phosphorylation; HFD, High-fat diet; ROS, Reactive oxygen species; OMM, Outer mitochondrial membrane; IMM, Inner mitochondrial membrane; MOMCs, Mitochondrial outer membrane channels; ETC, Electron transport chain; PGC-1α, Peroxisome proliferator-activated receptor gamma coactivator 1-alpha; UPRmt, Mitochondrial unfolded protein response; MMP, Mitochondrial membrane potential; MDVs, Mitochondrial-derived vesicles; TFAM, Mitochondrial transcription factor A; Nrf-1, Nuclear respiratory factor 1; Nrf-2, Nuclear respiratory factor 2; ERRα, Estrogen-related receptor alpha; mtDNA-CN, Mitochondrial DNA copy number; mtDNA, Mitochondrial DNA; SIRT1, Sirtuin 1; SIRT3, Sirtuin 3; AMPK, AMP-activated protein kinase; MAPK, Mitogen-activated protein kinases; eNOS, Endothelial nitric oxide synthase; Parkin, Parkin RBR E3 ubiquitin protein ligase; MD, Mitochondrial dynamics; Drp1, Dynamin-related protein 1; Opa1, Optic atrophy protein 1; Mfn1/2, Mitofusins 1/2; MFF, Mitochondrial fission factor; Fis1, Fission protein 1 ; ER, Endoplasmic reticulum; Cyt C, Cytochrome C; MMS, Mitochondrial misfolding stress; c-mtProt, Cytoplasm and mitochondrial protein precursors; HSP70, Heat shock protein 70; HSP60, Heat shock protein 60; ClpP, Caseinolytic protease; LONP1, Lon peptidase 1; PINK1, PTEN-induced kinase 1; LC3, Light chain 3; EVs, Extracellular vesicles; mPTP, Mitochondrial permeability transition pore; LA, Alpha-lipoic acid; SZ-A, Mulberry twig alkaloids; QG, Quercetagetin; MDA, Malondialdehyde; CA, Cholic acid; CDCA, Chenodeoxycholic acid; CYP7A1, Cytochrome P450 family 7 subfamily A member 1; CYP8B1, Cytochrome P450 family 8 subfamily B member 1; CYP27A1, Sterol 27-hydroxylase; CYP7B1, cytochrome P450 7B1 ; Ch25h, Cholesterol-25-hydroxylase; DCA, Deoxycholic acid; GCDCA, Glycochenodeoxycholic acid; UDCA, Ursodeoxycholic acid; BAs, Bile acids.
